# Inflationary theory of branching morphogenesis in the mouse salivary gland

**DOI:** 10.1038/s41467-023-39124-x

**Published:** 2023-06-09

**Authors:** Ignacio Bordeu, Lemonia Chatzeli, Benjamin D. Simons

**Affiliations:** 1 Department of Applied Mathematics and Theoretical Physics, Centre for Mathematical Sciences, University of Cambridge, Cambridge, UK; 2 Wellcome Trust/Cancer Research UK Gurdon Institute, University of Cambridge, Cambridge, UK; 3 Department of Physics, Facultad de Ciencias Físicas y Matemáticas, Universidad de Chile, Santiago, Chile; 4 Wellcome Trust-Medical Research Council Cambridge Stem Cell Institute, Jeffrey Cheah Biomedical Centre, University of Cambridge, Cambridge, UK

**Keywords:** Organogenesis, Computational biophysics, Computational models, Applied mathematics

## Abstract

The mechanisms that regulate the patterning of branched epithelia remain a subject of long-standing debate. Recently, it has been proposed that the statistical organization of multiple ductal tissues can be explained through a local self-organizing principle based on the branching-annihilating random walk (BARW) in which proliferating tips drive a process of ductal elongation and stochastic bifurcation that terminates when tips encounter maturing ducts. Here, applied to mouse salivary gland, we show the BARW model struggles to explain the large-scale organization of tissue. Instead, we propose that the gland develops as a tip-driven branching-*delayed* random walk (BDRW). In this framework, a generalization of the BARW, tips inhibited through steric interaction with proximate ducts may continue their branching program as constraints become alleviated through the persistent expansion of the surrounding tissue. This inflationary BDRW model presents a general paradigm for branching morphogenesis when the ductal epithelium grows cooperatively with the domain into which it expands.

## Abstract (web summary)

The authors show that the ramified ductal network of the mouse salivary gland develops from a set of simple probabilistic rules, where ductal elongation and branching are driven by the persistent expansion of the surrounding tissue.

## INTRODUCTION

In living organisms, branched ductal tissues and organs have evolved to optimize the exchange of nutrients, enzymes or waste products. To resolve the mechanisms that underlie the development and patterning of branched epithelia, emphasis has been placed on resolving transcription factors, signaling components and physical influences that instruct fate decisions and lineage selection at the cellular scale^1–4^. These studies have inspired the development of predictive coarse-grained theories that implicate the role of Turing-like processes in regulating the positioning and local dynamics of branch primordia during early phases of growth^5–7^. In particular, they have provided important insights into the mechanisms that drive the stereotypic patterns of growth that characterize many ductal epithelia such as lung and kidney. Complementary to these mechanistic studies, others have considered whether insight into branch patterning can be provided through the quantitative analysis of large-scale topologies of ductal trees. Here, some have proposed a “stereotypic” program of development operating at the ductal scale in which branching patterns emerge according to defined mathematical rules controlling delay patterns during serial rounds of dichotomous branching^8–11^. By contrast, others have argued in favor of a non-stereotypic branching program in which ductal networks develop according to a stochastic self-organizing principle based on the branching-annihilating random walk (BARW). In this model, proliferative cells localized at ductal tips, or endbuds, drive a serial process of ductal elongation and stochastic bifurcation that becomes terminated when active tips encounter secreted factors and/or steric influences from neighboring ducts. Applied to the mouse mammary gland epithelium, this minimal model predicts quantitatively a range of statistical features, from the localization and critical dynamics of active tips at the periphery of the expanding network, to the statistical organization and giant density fluctuations of the trailing ducts^12–16^. Applied to the secondary phases of branching morphogenesis, further studies have shown that the BARW model provides a faithful prediction of the branching statistics in the three-dimensional context of the mouse pancreas and kidney, as well as in cultured branching tissues, suggesting a conserved self-organizing principle^13,17–20^. Finally, recent studies have combined elements from these complementary descriptions to address whether and how these programs may integrate to drive the large-scale organization of branched ductal epithelia during later stages of development^21^.

Despite the success of the BARW as a model of branching morphogenesis, two features sit uncomfortably with experiment. In the mouse mammary gland, ductal tips grow into a preformed mesenchyme (known as the fat pad) that expands comparatively little during the (pubertal) phase of branching morphogenesis. Moreover, as tip cells contribute to the luminal and myoepithelial basal sub-lineages of the trailing ducts, they fall out of cycle, thereby not contributing further to the expansion of the ductal network. By contrast, in most developing branched epithelia, cell proliferation is not restricted to growing tips, but the network expansion occurs in concert with an expansion of the surrounding tissue, requiring the continuous remodeling of the ductal tissue. Here, applied to the development of the mouse salivary gland, we show that these factors conspire to undermine the validity of the simple BARW paradigm.

**Fig. 1:**
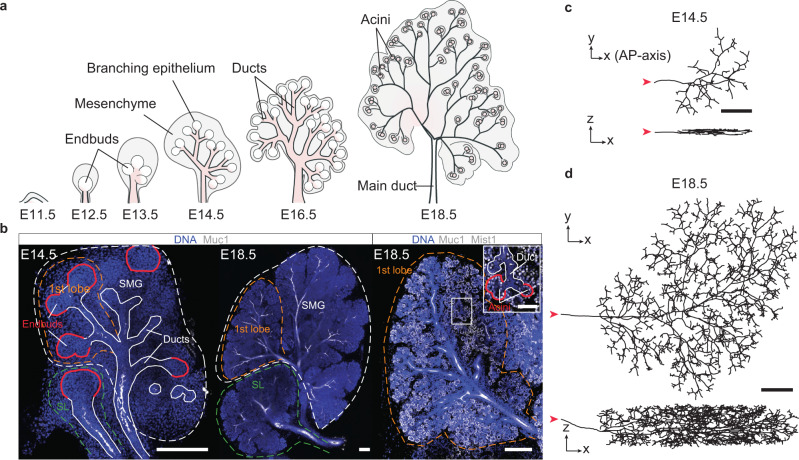
Development of the mouse salivary gland. **a** Schematic of salivary gland development depicting the cooperative expansion of epithelium and surrounding mesenchyme (adapted from^4^). **b** Confocal sections of the submandibular gland (SMG, white dashed) and sublingual gland (SL, green dashed) with 1st lobe (orange dashed) at E14.5 (left), E18.5 (center), and a close-up of the first lobe at E18.5 (right), stained with DAPI for nuclei (blue), Muc1 for ducts and nuclear Mist1 for acini (both white). In the first lobe, ducts (white) and endbuds/acini (red) are outlined in each panel. Top and side views of the skeletonized 3d reconstruction of the ductal network at (**c**) E14.5 and (**d**) E18.5, with the main duct identified by a red arrowhead whose orientation defines the AP-axis. For each time-point, *n* = 3 biological repeats were analyzed. All scale bars correspond to 200 μm.

The development of the mouse salivary gland begins at embryonic day (E)11.5 with a thickening and invagination of the oral epithelium (Fig. 1a)^22^. By E12.5, this process results in the formation of a stalk, a rudimentary duct whose endbud plays host to rapidly-cycling progenitor cells, whose fate specification if controled by a hierarchy of Kras and Notch signaling^4^. Around E13.5, the primitive endbud is partitioned into smaller endbuds in a coordinated process of cleft formation, which becomes reinforced through the accumulation of extracellular matrix proteins^23–25^. This process is illustrated at E14.5-E16.5 using a combination of explant live-imaging (Supplementary Figs. 1 and 2a and Supplementary Movie 1) and proliferation kinetics based on EdU incorporation (Supplementary Fig. 2b and Methods). Aided by a repulsive mechanism^26^, endbuds establish a sparse rudimentary branched structure from which subsequent expansion proceeds through a process of ductal elongation and tip bifurcation (Supplementary Fig. 2c), leading to the specification of a ramified ductal network (Fig. 1b). By E18.5, the branching process becomes terminated following the differentiation of tip cells into secretory acinar cells, that are surrounded by a layer of myoepithelial cells.

Here, by quantifying the statistical organization of the branched network during embryonic development, we show that the morphogenesis of the salivary gland network can be encapsulated within the framework of a generalized BARW model in which the cooperative expansion of the epithelium and the surrounding mesenchyme allows tips that become inhibited by steric influences to continue branching, ensuring that the network is distributed uniformly across the gland. As well as capturing the statistics of the branched network, we show that this minimal model, with parameters fully constrained by average properties of the network, can faithfully predict the non-stationary branch length distribution and the pattern of proliferation across the expanding network. We propose that this generalization, in which ductal expansion is tightly correlated with the growth of the surrounding tissue, may broaden the class of organs in which the basic BARW paradigm applies.

## RESULTS

### Branching organization of the developing mouse salivary gland ductal epithelium

**Fig. 2:**
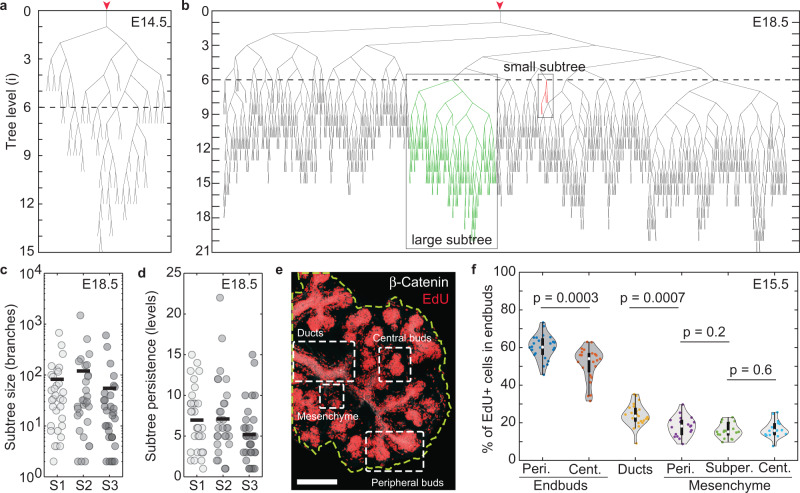
Branching organization of the mouse salivary gland. Reduced branching tree at (**a**) E14.5 and (**b**) E18.5. In (**b**), two subtrees are annotated with the common ancestor positioned at branch level 6 (dashed line). Dot-plot showing the heterogeneity of (**c**) subtree sizes (number of constituent ducts; average shown as bar) and (**d**) subtree persistence (highest level to which a subtree persists) for three biological repeats (S1, S2, and S3). **e** Cross-section of the center of an E15.5 SMG showing proliferative activity as indexed by short-term EdU incorporation and ducts visualized by *β*-catenin staining (Methods), scale bar corresponds to 200 μm. The outline of the gland is shown in green and regions of interest annotated. **f** Violin plots of EdU incorporation in different compartments: endbuds, ducts, mesenchyme, and regions: peripheral [Peri.], central [Cent.] and subperipheral [Subper.], of the gland at E15.5. Boxes denote 1st and 3rd quartiles with median point (white), whiskers mark the 5th and 95th percentiles, no points were considered outliers, (*p* values from two-sided t-tests). All data shown was obtained for *n* = 3 biological repeats.

To gain insight into the mechanisms that control the large-scale organization of the salivary gland, we dissected, stained and imaged in 3d murine glands using confocal microscopy (see Methods). Here, attention was placed on the submandibular gland (SMG), the most well-studied of the three major salivary gland compartments. Glands were stained with Mucin 1 (Muc1) to visualize the ductal lumen^27^, Mist1 to identify acini and DAPI to mark cell nuclei (Fig. 1b). From these images, we then manually segmented the entire 3d ductal network of the first lobe of the SMG at E14.5, E16.5, and E18.5, tracing the lumen through serial branch points down to individual endbuds/acini (Figs. 1c, d, 2a, b, Supplementary Figs. 2d–i, 3, 4 and 5, and Methods). This analysis revealed a small bias in growth along the anterior-posterior (AP)-axis (Supplementary Fig. 6a, b), as evidenced by a higher fraction of ducts that were aligned with the main duct. The 3d reconstructions showed a tendency for ducts to elongate and branch mostly parallel to the xy-plane (Supplementary Fig. 6c), consistent with the “pancake-like” morphology of the gland.

### Salivary gland shows a non-stationary growth characteristic

From 3d gland reconstructions, we then studied the topology of the ductal network at E18.5, when the branching process is complete (Fig. 2b, Supplementary Fig. 5, and Methods). In common with other branched organs, the results showed evidence of broad heterogeneity: From the ensemble of subtrees beginning at the 6th generation—termed level—of branching from the main duct (Fig. 2b), some were found to be small, terminating at low levels, while others were large, extending to level 20 or more (Fig. 2c, d). Such behavior was reminiscent of that observed in the mouse mammary gland^12–14^, where ductal morphogenesis has been associated with a BARW. Within this framework, the properties of the branching network depend on just two key parameters: the dimensionless ratio *r*_branch_ = *R*_branch_/*D*_*r*_, and the distance *ρ*_a_ that an oriented tip can approach a duct before becoming terminated (see Supplementary Note). Here, *R*_branch_ denotes the branch rate and *D*_*r*_ denotes the rotational diffusion constant of active ductal tips that undergo a persistent random walk (for details, see Supplementary Note). Based on this paradigm, we first questioned whether the statistical organization of the SMG network could be explained. With both parameters estimated empirically from the data (*r*_branch_ = 0.45 ± 0.02 and *ρ*_a_ = 36 ± 26 μm, Methods and Supplementary Note), we compared a range of branching characteristics with a stochastic simulation of the BARW model, using as further input the distribution of branching angles (Supplementary Fig. 6d and e) and the persistence length of ducts as measured at E18.5, the overall developmental time, and the rudimentary tree found at E14.5 (Supplementary Fig. 3) as the initial condition (for details, see Supplemental Note, section S1). However, the model could provide only a poor prediction of the network statistics (Supplementary Fig. 7, Supplementary Movie 2 and Methods).

**Fig. 3:**
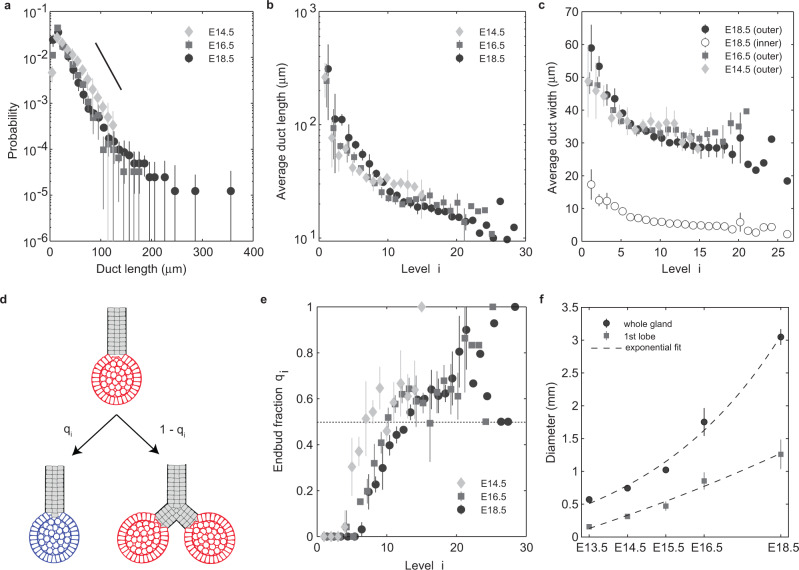
Morphometric measurements of salivary gland characteristics point to a growth dynamics that evolves over time. **a** Distribution of duct lengths at E14.5, E16.5, and E18.5; solid line indicates an exponential decay. **b** Average duct length as a function of branch level at E14.5, E16.4, and E18.5. **c** Average outer duct width as a function of branch level for each time point, and inner (luminal) diameter at E18.5. **d** Branching patterns may be characterized by a branch statistic in which, for each branch level *i*, a fraction *q*_*i*_ of ducts close with an endbud (blue), while the remainder undergo further bifurcation (red). **e** Endbud fraction *q*_*i*_ as a function of the branch level *i* at E14.5, E16.5, and E18.5. **f** Temporal variation of the diameter of the whole SMG and the first lobe, measured as the equivalent diameter of the 2d maximum intensity projection of the 3d confocal stack, during embryonic development. Dashed lines show an exponential fit with rates 0.014 h^−1^ (*R*^2^ = 0.94) and 0.0135 h^−1^ (*R*^2^ = 0.98), respectively. All plots show mean and SD from *n* = 3 biological replicates for each time-point.

The validity of the BARW as a model of branching morphogenesis relies implicitly on the “stationarity” of the branching process—that the ensemble of endbuds that remain active have the same proliferative *potential* throughout the branching process. However, consistent with a non-stationary branching process, measurements of proliferation kinetics based on EdU incorporation at the E15.5 time point showed that all cell compartments and, in particular, tip cells that lie within the center of the gland do not exit cycle but remain active, albeit with diminished proliferative index (Fig. 2e, f). We questioned whether this proliferative heterogeneity was accompanied by temporal changes in local branching characteristics that could compromise the integrity of the BARW model. By quantifying 3d network reconstructions at E16.5 and E18.5, we found that the distribution of duct lengths (defined as the distance between consecutive branch points) showed a “fat tail” that became longer with time (Fig. 3a), contrasting the robust exponential-like dependencies found for the mammary gland^12–14^. Further, measurements of the average duct length as a function of branch level showed the largest values at the lowest levels, with a continuous monotonic decrease with increasing level that became more pronounced during development (Fig. 3b). Similarly, the average (outer) duct widths were larger at the lowest generation numbers, with a tendency to plateau with increasing level (Fig. 3c), consistent with the reduction of endbud sizes with serial rounds of branching (Supplementary Fig. 2c).

To further characterize changes in the branching statistics, following ref. ^12^, we determined the branching probability as a function of generation number. Specifically, for each branch level *i*, we quantified the fraction *q*_*i*_ of ducts that close in an endbud rather than proceeding through a further round of bifurcation (cf. Fig. 3d). Notably, this analysis showed that, at E18.5, the endbud fraction *q*_*i*_ vanished over the first 5 generations (Fig. 3e). This contrasted with the behavior both at E14.5 and E16.5, where the endbud fraction became non-zero at a lower level index, with *q*_*i*_ staying larger than the E18.5 data at low branch levels (Fig. 3e). At the same time, the endbud fraction consistently decreased at most, if not all, levels between E14.5 and E16.5 and between E16.5 and E18.5. Together, these findings were consistent with a branching process that was not stationary, but evolved during development. We therefore questioned whether the morphogenesis of the mouse salivary gland may fit with a revised stochastic branching rule.

### The salivary gland develops as a branching-delayed random walk on an expanding domain

To identify a candidate model, we placed emphasis on the cooperativity of the developmental process: Notably, in contrast to the mouse mammary gland, salivary gland ductal development proceeds in concert with the expansion of the surrounding mesenchyme (Fig. 1a)^1,28–31^, with the latter providing both physical and chemical cues that support the branching process^32,33^. Indeed, measurements of the total gland size over the developmental time window revealed an exponential-like expansion between E11.5 and E18.5 (Fig. 3f), a behavior distinct from the boundary-driven linear-like radial expansion predicted by a BARW. Moreover, the persistent proliferation of ductal and tip cells throughout the gland (Fig. 2e, f) suggested that the expansion of tissue is not dominated by proliferation at the periphery, as would be expected in a BARW, but involves a uniform “inflation” of tissue in which trailing ducts expand in concert with the underlying mesenchyme. Such behavior would provide a qualitative, if not yet quantitative, explanation for the seemingly non-stationary character of the duct length distribution: Ducts formed early in development, at low generation numbers, have longer to expand than those born late. Moreover, while steric influences from neighboring ducts could lead to the growth inhibition of endbuds that become submerged within the ductal network, away from the periphery, the expansion of tissue may alleviate these constraints enabling branching to continue (see Supplementary Fig. 1). Such behavior would be consistent with the observed cell proliferation seen in both the epithelium and mesenchyme across the gland (Fig. 2f). It would also explain why submerged endbuds remain proliferatively active during development and, through their continual branching, how the fraction of endbuds at low generation numbers can fall between E14.5 and E18.5 (Fig. 3e).

**Fig. 4:**
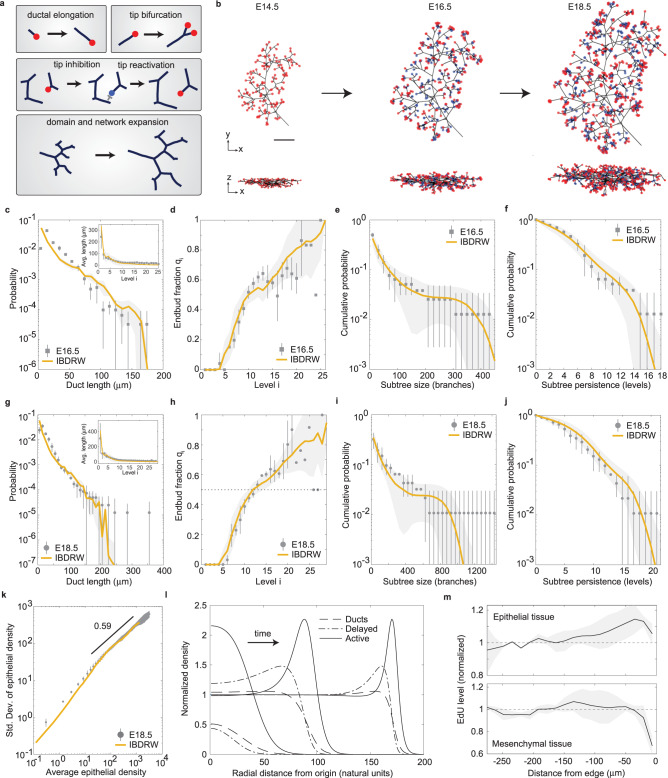
Salivary gland ductal epithelium organizes as an inflationary branching-delayed random walk (IBDRW). **a** Schematic of sub-processes in the IBDRW model. **b** Stochastic simulation of the 3d IBDRW model from E14.5 (initial condition) to E16.5 and E18.5, showing a front (top) and lateral (bottom) view, with ducts (black), active (red), and delayed (blue) tips (see Methods and Supplementary Note, section S1). Scale bar 200 μm. **c–f** Comparison of the experimental E16.5 and **g–j** E18.5 data (mean and SD, *n* = 3 biological replicates for each time point) and simulations (line and shaded SD, *n* = 10^3^ realizations) of the (**c**, **g**) endbud fraction *q*_*i*_, the cumulative distribution of (**d**, **h**) subtree persistence and (**e**, **i**) subtree sizes, and (**f**, **j**) the cumulative distribution of duct lengths and (inset) average duct length as a function of level index. **k** Giant density fluctuations of the ductal epithelium for E18.5 data (mean and SD, *n* = 3 biological replicates) and IBDRW model (yellow line, *n* = 10^3^ repeats). **l** Density profile of active and delayed tips, and inactive ducts at different times in the co-moving frame of the expanding 2d system as inferred from MFT (Supplementary Note Eq. (S16)), with ${r}_{{{{{{{{\rm{branch}}}}}}}}}/{r}_{\exp }=8$ (see Supplementary Note for details), and densities normalized by the corresponding value of the homogeneous steady-state solution. **m** Quantification of EdU incorporation as a function of the distance from the periphery of the lobe at E15.5 (mean and SD, *n* = 3 biological replicates) for epithelial (top) and mesenchymal tissue (bottom) (cf. Fig. 2e and Methods). Intensities are normalized to the average intensity around the center of the lobe.

Based on these findings, we questioned whether the branching dynamics of the salivary gland could be captured by a generalization of the BARW paradigm in which the dynamics of the growing ductal epithelium undergoes an inflationary branching-*delayed* random walk (IBDRW): In this model, branching morphogenesis takes place in the context of an expanding domain in which the proliferation of the ductal epithelium and surrounding mesenchyme are correlated to ensure that the connectivity of the ductal network is maintained over the developmental time course (see Fig. 4a). In the context of the salivary gland epithelium, this requires that, as tissue expands, cells must proliferate and ducts elongate with the same exponential rate constant. While a direct quantitative comparison of proliferation rates in the ductal epithelium and mesenchyme is compromised by the unknown fate behaviors of the consistent cell populations, we note that, based on EdU incorporation (Fig. 2f), both compartments do indeed show a similar proliferative index. However, on the question of whether ductal elongation is slave to the growth of the surrounding mesenchyme, as seems likely, or whether the reverse is true, as long as the two compartments grow in concert, the model is agnostic.

To test the validity of the IBDRW model, we placed emphasis on measurements of the endbud fraction (Fig. 3e) and the distributions of subtree size and persistence. Here, a subtree was defined as a clade of the branching network that had a last common ancestor at level 6 (see Fig. 2b). This value was chosen as it represents approximately the lowest level of branching at which terminated endbuds (or acini) are first observed in the E18.5 data. From these results, we could determine the size as the total number of ducts in the subtree and persistence as the maximum number of levels spanned by a given subtree. Within the framework of the IBDRW model, the statistics of the salivary gland branched network depend now on a third key parameter: alongside the ratio *r*_branch_, and the inhibition radius *ρ*_a_, there is the rate of exponential expansion scaled by the rotational diffusion constant, ${r}_{\exp }$ (see Supplementary Note for details).

Using an agent-based representation of the IBDRW (Fig. 4b and Supplementary Movie 3), we then made a least-squares fit of the model, integrating measurements of the endbud fraction and distributions of subtree size and persistence at E18.5 and using as further input the measured value of *r*_branch_ = 0.45, the distribution of branching angles (Supplementary Fig. 6d, e), the duct persistence length, and time span of the branching process. This left just two adjustable fit parameters, *ρ*_a_ and ${r}_{\exp }$, both of which were heavily constrained by the experimental data. (For details of the stochastic simulations and list of parameters, we refer to the Supplementary Note.) Taking as an initial seed the measured spatial network distributions at E14.5, sampled from three glands, the model provided a good fit to all three distributions with *ρ*_a_ = 45 μm and ${r}_{\exp }=0.054$, both at E16.5, when the branching process is not yet complete (Fig. 4c–e, *R*^2^ = 0.88, *R*^2^ = 0.96 and *R*^2^ = 0.99, respectively), and E18.5, when the branching process has terminated (Fig. 4g–i, *R*^2^ = 0.84, *R*^2^ = 0.95 and *R*^2^ = 0.97, respectively). (A sensitivity analysis of the fits is shown in Supplementary Fig. 9 and discussed in the Supplementary Note.) With these parameters, the model could also predict well the decay of the duct length distribution, including the abundance of “over-sized” ducts, as well as the change in average duct length with branch level at E16.5 (Fig. 4f, *R*^2^ = 0.07, ${R}_{\log }^{2}=0.80$ and inset *R*^2^ = 0.90) and E18.5 (Fig. 4j, *R*^2^ = 0.55, ${R}_{\log }^{2}=0.75$ and inset, *R*^2^ = 0.92).

We then quantified the ductal density fluctuations, which provide an indication of how effectively the ductal network fills space. Previously, in the context of the mammary gland, this was achieved by quantifying the local epithelial density across the fat pad^13^. By plotting the estimated standard deviation in the epithelial volume as function of the average at E18.5 (Fig. 4k), we found convergence to a statistical scaling behavior for both the experimental data and the IBDRW model, with an exponent of around 0.59 (see Methods and Supplementary Note), close to the uncorrelated value of 0.5. This compares to the value of 0.66 found for the mammary gland^13^, and may reflect differences in the nature of the dynamics that allows submerged tips in the salivary gland to continue branching and contribute to regions that would be otherwise inaccessible.

To further challenge the predictions of the IBDRW model, we considered whether the hallmark statistical distributions of the branching salivary gland network could be recapitulated in the context of an ex vivo culture system. Optimizing culture conditions, we found that explants harvested at E14.5 could be grown and imaged in time-lapse every hour over a 2–3 day time window (Supplementary Movie 1). By quantifying the spatial organization of the 3d branched network following 40 hours of time-lapse imaging, we found a variable degree of branching with some tips undergoing as many as 4 rounds of branching while others did not branch at all (Supplementary Fig. 1). Notably, endbuds on the periphery branched more readily than those remote from the boundary (Supplementary Fig. 1). Segmentation and analysis of the full ductal network of explants after 3 days of culture (see Supplementary Fig. 10) revealed a striking similarity of the tree statistics with those of the E18.5 in vivo gland (Supplementary Fig. 11), despite being effectively one day younger; a result confirmed by stochastic simulation of the IBDRW model. However, the explants showed slightly higher endbud fractions at low branch levels and slightly smaller subtrees compared to the E18.5 in vivo gland (see Supplementary Fig. 11), consistent with their lower developmental age. Together, these results show that, under explant conditions, the salivary gland follows a similar pattern of branching morphogenesis, albeit with an expansion rate that is accelerated potentially due to the relaxation of spatial constraints.

Finally, we compared the predicted branching statistics across the developmental time course when seeded as a single stalk. In this case, a reasonable quantitative comparison could only be found by fine-tuning the model parameters away from those estimated experimentally. In this context, stereotypic features of the true branching process, including the early phase of endbud diversification through parallel cleft formation, leads to small but statistically significant departures from the predicted distributions, compromising the validity of the IBDRW model.

Altogether, these findings support the hypothesis that, during the late phase of branching morphogenesis, the large-scale organization of the ductal network in the salivary gland emerges from a local statistical rule in which steric influences reduce the rate of cell proliferation and bifurcation of submerged tips, until the cooperative expansion of the epithelium and surrounding tissue alleviates the constraint (see Supplementary Fig. 7).

### Mean-field theory of the IBDRW model

Although stochastic simulations of the IBDRW provide a platform to address the spatial organization and statistics of the branching network, and make comparison with experiment, it does not provide analytical insights into dynamics of the branching process. To determine how average properties of the developing network depend on key parameters of the model, we turned to a coarse-grained or mean-field analysis. Previously, studies of the BARW model using field theory methods placed the system in the universality class of general epidemic processes^34,35^. There, the BARW was interpreted as an epidemic process, where proliferatively active tips function as “infectious agents”, *A*, that multiply (tip bifurcation) and diffuse. As they move, they leave behind a trail of “infected sites”, *I*, (inactive ducts) that become toxic, so that should they re-encounter these sites they become annihilated (tip termination). Formally, as a function of the relative branch rate, *r*_branch_, the BARW model shows a transition from a phase in which networks terminate fully with probability one, to a phase in which the probability of indefinite network expansion becomes non-zero. While the critical dimension of the BARW model is 6^34^, far from the critical point the evolution of tip and duct densities can be addressed within the framework of mean-field theory (MFT)^13^.

In the present case, the branching dynamics is not stationary, but takes place on an expanding domain in which inactive ducts grow in concert with the underlying mesenchyme. Moreover, when active tips *A* encounter ducts *I*, they do not terminate but are inhibited or slowed, effectively entering a “delayed” state, *S*, becoming reactivated when space is opened up. At the level of MFT, the dynamics of the *d*-dimensional IBDRW model is defined by the kinetic equations

1 $$	\frac{\partial A}{\partial t}+{{{{{{{\bf{u}}}}}}}}\cdot \nabla A+d{R}_{\exp }A=D{\nabla }^{2}A+{R}_{{{{{{{{\rm{branch}}}}}}}}}A-{R}_{{{{{{{{\rm{delay}}}}}}}}}A(A+I+S)+{R}_{\exp }S\\ 	 \frac{\partial S}{\partial t}+{{{{{{{\bf{u}}}}}}}}\cdot \nabla S+d{R}_{\exp }S={R}_{{{{{{{{\rm{delay}}}}}}}}}A(A+I+S)-{R}_{\exp }S\\ 	 \frac{\partial I}{\partial t}+{{{{{{{\bf{u}}}}}}}}\cdot \nabla I+d{R}_{\exp }I={R}_{\exp }I+{R}_{{{{{{{{\rm{duct}}}}}}}}}A,$$

where *A*(**x**, *t*), *I*(**x**, *t*) and *S*(**x**, *t*) denote, respectively, the local density of active tips, ducts and delayed tips. To trace the dynamics of the growing tissue, these equations must be solved subject to a no-flux boundary condition with *A*(**x**, *t*) = *A*_0_*δ*(**x**)*δ*(*t*) and *S*(**x**, *t* = 0) = *I*(**x**, *t* = 0) = 0 (Supplementary Note). Here, *D* denotes the diffusion constant, *R*_branch_ the branch rate, *R*_duct_ the rate at which ducts are created by active tips, *R*_delay_ the delay rate, and ${R}_{\exp }$ the expansion rate of the gland. To simplify the analysis, we have approximated the persistent random walk of active endbuds as a simple diffusive process. Although more complex dynamics may be introduced at the MFT level^36^, such refinements would not effect the long-term steady-state dynamics of the expanding tissue. Moreover, at the mean-field level, the effect of the inhibition radius can be subsumed into the effective delay rate, *R*_delay_.

When ${R}_{\exp }=0$, Eqs. (1) collapse to those studied in the context of the BARW^13,14^. However, in the expanding system (${R}_{\exp } > 0$) new terms appear: Alongside the last set of terms on the right-hand side of the equations that reflect, respectively, the expansion-driven reactivation of delayed tips and the linear growth of inactive ducts, the inflation of the domain generates an advective term proportional to ${{{{{{{\bf{u}}}}}}}}({{{{{{{\bf{x}}}}}}}},t)={R}_{\exp }{{{{{{{\bf{x}}}}}}}}$, and a dilution term proportional to $d{R}_{\exp }$^37,38^. Rescaling the rates by *R*_duct_ and time by ${R}_{{{{{{{{\rm{duct}}}}}}}}}^{-1}$, space by ${(D/{R}_{{{{{{{{\rm{duct}}}}}}}}})}^{1/2}$, defining the dimensionless flow field ${{{{{{{\bf{v}}}}}}}}={{{{{{{\bf{u}}}}}}}}/{({R}_{{{{{{{{\rm{duct}}}}}}}}}D)}^{1/2}$, and introducing the rescaled density fields *a* = *AR*_delay_/*R*_branch_ (similarly for *S* and *I*), the kinetic equations (1) may be written in dimensionless form, identifying just two dimensionless parameters, *r*_branch_ and ${r}_{\exp }$ (see Eq. (S15) in Supplementary Note).

Analyzes of the mean-field equations predict that, in two dimensions and above, the IBDRW model has two homogeneous solutions *ρ* = (*a*, *s*, *i*): an inactive state, *ρ*_0_, where the density of tips and ducts vanish (measure zero), and an active state *ρ*_+_, where all densities are non-zero (Supplementary Note). When the branching rate is higher than the volumetric expansion rate (${r}_{{{{{{{{\rm{branch}}}}}}}}} > \, d{r}_{\exp }$), the empty state *ρ*_0_ is unstable and an initial “pulse” of active tips is predicted to grow and eventually populate the entire system (Fig. 4l). When ${r}_{{{{{{{{\rm{branch}}}}}}}}} < \, d{r}_{\exp }$, the domain expands at a faster rate than the ductal network, so that the density of ducts and tips decreases steadily, converging towards the empty state *ρ*_0_. Physically, this represents a dilute limit, where ductal formation and branching events are too infrequent to compensate for the expansion of the gland.

In the framework of the BARW (${r}_{\exp }=0$), the mean-field equations assume the form of a Fisher-KPP reaction-diffusion system in which active tips localize as a soliton front that propagates at constant speed $v=2\sqrt{{r}_{{{{{{{{\rm{branch}}}}}}}}}}$, leaving behind a constant density of inactive tips^13^. In the active state of the IBDRW model, when ${r}_{{{{{{{{\rm{branch}}}}}}}}} > d{r}_{\exp }$, the expanding front of active tips becomes advected by the expanding domain on which it grows. Transforming to the frame of the exponentially expanding tissue, numerical integration of the mean-field equations show that the relative speed of this front gradually diminishes to zero. While the expanding front shows an enrichment of active and delayed tips, the trailing density of all three components converges to constant values, consistent with the persistent reactivation of delayed tips as space is created through expansion (Fig. 4l). Although the mean-field rates are effective parameters that do not correspond straightforwardly to their microscopic counterparts (see Supplementary Note), the ratio ${r}_{{{{{{{{\rm{branch}}}}}}}}}/{r}_{\exp }$ is conserved (as long as both quantities rescale identically). It therefore follows that, with the experimental fits at *r*_branch_ ≈ 0.45 and ${r}_{\exp }\approx 0.054$, the system is placed in a regime where space becomes filled with a uniform density of ducts and tips.

Consistent with the profile of densities predicted by the IBDRW model, measurements of the proliferative index of the ductal epithelium at E15.5 (Fig. 4m, top panel) showed an enrichment of the proliferation marker EdU at the periphery of the gland, with an approximately constant level in the bulk (cf. Fig. 2f). Furthermore, quantification of the proliferation index of the mesenchyme (Fig. 4m, bottom panel) revealed a uniform distribution of proliferative cells, as expected for a uniform (exponential) inflation of tissue.

## DISCUSSION

Recent studies of the large-scale statistical organization of ductal networks have placed emphasis on stochastic models in which the development of the branched epithelium has been considered as decoupled from the dynamics of the surrounding mesenchyme. However, in most branched epithelia, ducts and mesenchymal tissue expand in concert^1,2^, with the growth of the former constrained by changes in the latter. In the salivary gland, these changes are accompanied by a constant remodeling of the epithelium in which ducts expand through cell proliferation, and tips inhibited by steric influences are able to continue branching as space becomes available. By developing a generalization of the BARW model of branching morphogenesis, we have shown that the statistical properties of the growing salivary gland network can be explained within the framework of a minimal model, where a local probabilistic branching rule is supplemented by constraints imposed by the global growth characteristic of the tissue.

Based on these findings, it makes sense to question whether the IBDRW model might apply to the development of other ductal tissue types. Notably, previous studies have shown that the statistical organization of the ductal network of mouse kidney during the late embryonic phase of development (E15–E19) can be explained within the framework of a conventional BARW^13^. Yet, in common with the salivary gland, branching morphogenesis in the kidney also takes place in the context of an expanding mesenchyme, with all ductal endbuds remaining proliferatively active during this phase, questioning the basis of the success of the BARW model. However, in contrast to the mouse salivary gland (Fig. 3f), the growth of the kidney during this period is not exponential, but linear-like (Supplementary Fig. 8a). How would the behavior of the IBDRW model differ if the tissue followed a linear rather than exponential growth characteristic? In a linear growth regime, the branching system would eventually converge towards a state in which submerged tips would have to arrest fully (i.e., experience a temporal pause) before resuming their growth as space became available (see Supplementary Note). In this regime, the network contribution arising from the reactivation of submerged tips becomes subleading, with the dominant contribution arising from tips positioned at the periphery of the gland (Supplementary Fig. 8b and Supplementary Note, section S4). As a result, it may be difficult to discriminate between the dynamics of a BARW and an IBDRW based on branch statistics alone. By contrast, when the expansion is exponential, in the long-term regime, tips never fully arrest, but their advance is only delayed by steric influences, with submerged tips continuing to contribute significantly to the network expansion. Now, in the case of the *first lobe* of the salivary gland, our quantifications of gland size fit equally well with an exponential or linear-like growth characteristic (Fig. 3f). Yet, by quantifying the dynamics in this crossover phase between linear and exponential, we have seen that the IBDRW provides a better fit to the branch characteristics than the BARW.

Finally, while the generalized IBDRW model predicts the statistical organization of the SMG branched network, it does not explain the processes that regulate the sustained (exponential-like) expansion of gland, nor the factors that ultimately bring about the growth termination and differentiation of tips into acini. However, this extended model may provide a quantitative platform to explain the pattern of branching morphogenesis in other ductal tissue types, laying the foundation to develop insights into the molecular programs that mediate branching and cell-fate specification.

## METHODS

All experiments were conducted at University of Cambridge, comply with the Home Office regulations, and were approved by the University of Cambridge Animal Welfare and Ethical Review Body.

### Mouse models

The *Rosa26-CreERT2*; *Rosa26-Confetti* line was used for ductal reconstruction^39–41^, *mTmG* mice were used for live-imaging^42^, and wild type C57BL/6J were used for immunofluorescence staining with laminin and EdU. The *Rosa26-CreERT2*; *Rosa26-Red2KrasG12D* mouse^43^ was used to compare the branching network of lobe 2 and 4 with the confetti lobe 1 (Supplementary Fig. 12).

All mice were maintained on a C57BL/6 background. The embryonic stages used were of E14.5, E16.5, and E18.5. Adult females of 6–20 weeks of age and males of 6–32 weeks of age were used for breeding. Since there is no sexual dimorphism at the embryonic stage, we did not distinguish between males and females.

### Tissue preparation

Submandibular glands were dissected from timed mating pregnant females. Day 0 was the day of the vaginal plug. To maintain the tissue integrity, the whole capsule containing the submandibular and the sublingual gland was isolated as previously described and processed for either fixation or explant culture^44^. The time of fixation varied depending on the stage. E13.5 to E15.5 salivary glands were fixed for 30 min in 4% Paraformaldehyde shaking at room temperature. E16.5 to E18.5 were fixed for 1 h at the same conditions.

### Explant cultures

Salivary glands were grown as explants for a short term period for either EdU staining or to prepare for live-imaging. Explants were cultured as previously described^45^. Briefly, salivary glands were dissected at E15.5 and cultured on a 0.4 μm pore filter (Falcon) floating on Advanced Dulbecco’s Modified Eagle Medium F12 (Advanced DMEM/F-12, Gibco®) supplemented with 1% GlutamaxTM (Gibco®), 1% penicillin–streptomycin (Sigma-Aldrich), 150 μg/ml ascorbic acid and 50 μg/ml transferrin at 37 °C and 5% CO_2_.

### EdU staining

Dissected E15.5 salivary glands were cultured as explants and treated immediately with 30 μM EdU (Thermo Fisher Scientific) diluted in Advanced DMEM;F12 with 1% penicillin-streptomycin and 1% Glutamax for 1 h. Salivary glands were then fixed for 30 min and stained for EdU as whole mounts using the EdU Click-iT Imaging Kit (Life technologies) according to the manufacturer’s protocol with the following modifications: the permeabilization step was extended overnight using 1% Triton-100X diluted in PBS while the staining step was extended to 4 h.

### Quantification of percentage of EdU^+^ cells

From EdU stainings at E15.5 (Fig. 2e and Supplementary Fig. 2b), we manually quantified the fraction of cells that were EdU^+^ from *n* = 3 samples (Fig. 2f), in different regions and compartments of the tissue. Endbuds were grouped into those positioned peripherally and centrally. Peripheral endbuds corresponded to the subset of endbuds that were located in direct proximity to the lobe boundary, while all other endbuds were considered as central. A similar distinction was made for the mesenchyme, which were grouped into peripheral, sub-peripheral (those just underneath the peripheral region) and central. Ducts, as opposed to endbuds, were not separated into groups.

### Quantification of radial EdU levels

From EdU stainings at E15.5 (Fig. 2e), the density of EdU incorporation was quantified computationally from *n* = 3 samples in 100 μm thick sections along the center of the lobe, as a function of the distance from the boundary of the tissue. By tracing the boundary of tissue (dashed line in Fig. 2e), we determined the average EdU intensity of pixels located between *r* microns and *r* + *dr* microns away from the boundary of the tissue, with *dr* = 25 μm and *r* = 0,  25,  $\ldots$  ,  200,  225 μm. The average EdU intensity in each stripe was normalized by average EdU intensity at *r* = 225 μm.

### Immunofluorescence of whole mount salivary glands

Fixed salivary glands were first permeabilised in 0.5% Triton 100 × in PBS for 4 h and then blocked for 1 h in 2% donkey serum and 0.5% Triton-100X diluted in PBS (blocking solution). After blocking, salivary glands were incubated with the primary antibody diluted 1:100 in the blocking solution. E13.5 to E15.5 glands were incubated for 4 days shaking at 4 °C, while E16.5 to E18.5 were incubated for 7 days shaking at 4 °C. For the secondary antibody staining, salivary glands were first washed six times for 1 h in 0.5% Triton-100 × PBS at room temperature to remove the primary antibody. Then salivary glands were incubated with the secondary antibody diluted 1:500 in blocking solution for 5 days, shaking at 4 °C. Following the incubation, the tissue was washed six times for 1 h in 0.5% Triton-100 × PBS at room temperature and mounted on 22 × 32 mm coverslips with a 0.25 mm i-spacer (Sunjin Lab) in RapiClear 1.52 (SunJin Lab). The mounted tissue was incubated overnight at 4 °C to allow clearing of the samples and then imaged on the confocal microscope. The primary antibodies used were rabbit anti-laminin (L9393, Sigma), rabbit anti-Muc1 (ab15481, Abcam), anti-Mist1 (ab187978, Abcam) and *β*-catenin (L54E2) Alexa Fluor 488 Conjugate (#2849, Cell Signalling). The secondary antibody used was Alexa Fluor 647 Donkey Anti-Rabbit (A31573, Thermo Fisher Scientific).

### Live-imaging

Short-term live-imaging of the salivary glands explants (Supplementary Fig. 2a) was performed as previously described^33^ on a Zeiss 880 Airyscan inverted confocal with the following modification. 50% Matrigel (Corning) was added on top of the nuclepore filter after adhering the filter with the sample into the double adhesive imaging spacers (Thermo Fisher Scientific). Images were taken with the 25× water immersed objective, every hour for a period of 15 h. Explants were maintained at 37 °C and 5% CO_2_.

Long-term live-imaging of salivary gland explants were performed using the Nikon SoRa inverted spinning disk confocal. Salivary glands were dissected at E14.5 and cultured as described in “Explant cultures”, on six-well glass bottom plates. Images were acquired every hour for 2 days (Supplementary Fig. 1 and Supplementary Movie 1).

### Imaging and image analysis

Fixed salivary glands were imaged using the Leica SP8 X White Light laser confocal microscope. Images were mainly obtained with a 20 × oil-immersed objective and processed with Fiji (ImajeJ2 v2.9.0/1.53t)^46^. For 3d ductal reconstruction, images of the whole first lobe were acquired with a 20 × oil-immersed objective and then stitched together using the LAS software v2.8.0, Leica. The tracing of the ductal network was performed manually using a purpose-built graphical user interface, implemented in MATLAB (R2022b, The MathWorks, Inc., Natick, Massachusetts, USA). To make Supplementary Movie 1 the z-stack from long term live-imaging was stabilized during post-processing using the StackReg plugin^47^ in Fiji.

### Reconstruction of the branching tree

The ductal network of the first lobe of the SMG was reconstructed at E14.5, E16.5, and E18.5 for *n* = 3 biological replicates in each case. This allowed us to trace the lumen of ducts from the main duct, through bifurcations, until reaching the endbuds, resulting in a 3d reconstruction of the ductal network (Supplementary Figs. 3, 4, and 5). Alongside the spatial organization of the ductal tree, we recorded both the outer diameter of each duct in the tree and, where possible, the inner (luminal) diameter (Figs. 3c and Supplementary Fig. 2d). The lumen was nonexistent at E14.5 and only partially developed at E16.5. We therefore only recorded the inner (luminal) diameter of ducts at E18.5. Using the same approach, the ductal network of lobes 2 and 4 of the SMG was reconstructed at E18.8 for *n* = 2 biological replicates using red2Kras confetti mice induced at low (clonal) density (see Supplementary Note and Supplementary Fig. 12). Finally, the ductal network of the first lobe of the *n* = 4 SMGs harvested from E14.5 *mTmG* embryos was reconstructed after culturing and live-imaging as explants for 3 days (Supplementary Fig. 10).

The branching tree was then reduced by representing the ductal network as a series of dichotomous bifurcation and termination events. In network theory language, this corresponded to an unweighted branching tree whose nodes have either degree 1, corresponding to termination points, or degree 3, corresponding to bifurcation events. Here, we ignored duct lengths, which allows the analysis to be focused on the topology of the network rather than on their spatial properties. In the event of a trifurcation—a branching event where a tip appears to split into tree branches—we artificially split them into two consecutive dichotomous bifurcations with a duct of length of zero separating them. These two representations are effectively indistinguishable. Yet, doing so eases the comparison with the model while not affecting the quantitative predictions for the properties of the branching tree (see Supplementary Note).

### Endbud fraction, *q*_*i*_

To characterize the branching statistics at different developmental time points, we measured the endbud fraction, *q*_*i*_, at level *i* defined as the fraction of endbuds found at level *i* compared to the total number of ducts at that level (Fig. 3d). Note that, at intermediate times (e.g., E14.5 and E16.5), a branch terminating in a endbud may undergo further branching at later times.

### Subtree size and persistence

For a given branching tree, subtrees were obtained by removing all nodes on or below level 5. This creates a set of disconnected subtrees, whose common ancestors all lie at level 6. For a given subtree, its size corresponds to the total number of ducts from which it is comprised, and its persistence corresponds to the maximum number of levels that it spans. With these measures, we could compute the cumulative probability of subtree sizes and persistence. Given a probability distribution *f*(*x*) of a random variable *x* (size or persistence), the (complementary) cumulative probability is defined as ${\bar{F}}_{X}(x)=f(X > x)=1-f(X\le x)$, and gives the probability that an event *x* is larger than a given value *X*.

### Statistics and reproducibility

No statistical method was used to predetermine sample size. No data were excluded from the analyses; the experiments were not randomized; the Investigators were not blinded to allocation during experiments and outcome assessment.

### Reporting summary

Further information on research design is available in the Nature Portfolio Reporting Summary linked to this article.

## Supplementary information

Supplementary Information

Description of Additional Supplementary Files

Supplementary Movie 1

Supplementary Movie 2

Supplementary Movie 3

Reporting Summary

## Data Availability

Source data are provided as a Source Data file. Source data are provided with this paper.

## Source data

Source Data

## Code availability

Scripts for the agent-based simulations of the IBDRW model and numerical integration of the mean-field equations are available on the following Github repository: https://github.com/ibordeu/scripts_IBDRW_salivary_gland_git.
