# Supplementary material for: Inflationary theory of branching morphogenesis in the mouse salivary gland
Source: Nat Commun. 2023 Jun 9;14:3422. doi: 10.1038/s41467-023-39124-x (PMC10256724; doi:10.1038/s41467-023-39124-x)
Supplement: Supplementary file 1 — Supplementary Information [file 41467_2023_39124_MOESM1_ESM.pdf]

Supplementary Information

Inflationary theory of branching morphogenesis in the mouse salivary gland

Here we develop in more detail the computational and theoretical framework used to study the branching dynamics of the salivary gland ductal epithelium. First, we define the agent-based implementation of the stochastic IBDRW model, and how the results of stochastic simulations were compared and fit to the experimental data. We then turn to the construction of the mean-field approximation of the IBDRW model, which we use to gain analytic insight into the growth dynamics of the branching process. Finally, we discuss in brief the potential application of the IBDRW to other branching contexts.

S1. STOCHASTIC SIMULATIONS OF THE IBDRW MODEL USING AN AGENT-BASED REPRESENTATION

To investigate the capacity of the BARW and its generalization, the IBDRW model, to predict the statistical distributions of the salivary gland network, we implemented a computational agent-based model of the stochastic branching process. The IBDRW comprises a set of processes illustrated in Fig. 4a, which include stochastic tip propagation, duplication (tip bifurcation), delay and reactivation, which all take place on the background of a global, constant, inflation of the domain on which the dynamics takes place. From the biological perspective, this means that, during development, ducts must expand through cell proliferation to match the tissue expansion. Therefore, in the context of the salivary gland, the distance between any two distinct points on the network necessarily increases exponentially over time (see Figs. 1 and 2f).

To simulate the full stochastic dynamics of the IBDRW model, we considered an off-lattice, discrete time, agent-based implementation. The dynamics of the conventional BARW model follows the same program but in the absence of domain expansion. To capture the dynamics, three particle species were considered: active tips (endbuds), immobile tracer particles (representing the locus of trailing ducts), and delayed tips (endbuds partially inhibited by local steric constraints). Active tips were subject to two concurrent Poisson processes: tip branching and propagation.

Formally, at each time-step of fixed duration Δt, we determined the number, $a(t)$, of active particles at time t. We then constructed a list of uniformly distributed random numbers r_i on the interval $[0, 1]$, with $i = 1, ..., a(t)$, and cycled through all active particles in random order, allowing them to either branch or "hop" in the manner of a persistent random walk. A particle j underwent branching if the branch probability $r_j < p_{\mathrm{b}} = (1 - \exp(-R_{\mathrm{branch}}\Delta t))$ (with R_{branch} the constant branch rate), or otherwise hopped. Branching was implemented non-locally such that, when a particle branched, it was removed and replaced by two active tips at new locations.

Following the propagation of an active tip, an inactive particle was left behind, representing the trail of elongating ducts. Active particles became delayed when they came within an inhibition radius, ρ_{a}, of a tracer particle. We also applied a "sensing cone" such that an active particle was only inhibited by tracers laying within a spherical sector around its direction of motion. This had two purposes: first, this prevented the (non-physical) immediate inhibition of active tips due to their interaction with their own proximate trailing duct; and, second, this ensured that inhibition did not immediately occur between two newly-born tips, but was rather due to direct tip-duct interactions. (More generally, in systems where inhibition occurs in response to biochemical signals secreted from maturing ducts, tips may sense chemical diffusion gradients coming from any direction. However, here, we focused on a model in which tips are inhibited by steric influences alone.)

The ductal network of the salivary gland develops as a three-dimensional tissue. We therefore considered the tip growth dynamics as a 3d persistent random walk [1], with zero translational diffusion but non-zero rotational diffusion constant D_r. Given the 3d position $\mathbf{r}_i(t) = (x_i, y_i, z_i)$ of an active tip at time t, with a director oriented according to the polar and azimuthal angles (θ, ϕ) in spherical coordinates, an elongation event implied a translation of the active tip from position $\mathbf{r}_i(t)$ to position

$$\mathbf{r}_i(t + \Delta t) = \mathbf{r}_i(t) + \Delta \mathrm{L}(\sin\theta' \cos\phi', \sin\theta' \sin\phi', \cos\theta'), \tag{S1}$$

where the step length, $\Delta L = V_0\Delta t$, corresponds to the displacement of a tip, moving at a speed V_0, during the

time-step Δt, and

$$(\theta', \phi') = (\theta, \phi) + \sqrt{2D_r\Delta t}\left(\frac{\arccos(1-2u_1)}{\pi} - 1/2, u_2\right). \tag{S2}$$

Here, the second term on the right-hand side corresponds to a uniformly distributed noise on a 3d sphere, with $u_1 \in U([0,1[)$ and $u_2 \in U([-1,1])$, with amplitude $\sqrt{2D_r\Delta t}$. The angular diffusion constant D_r encodes the persistence time of ductal growth. In the absence of noise, $D_r = 0$, ducts grow along a straight line, exhibiting infinite persistence. However, for $D_r > 0$, ducts diffuse with $\tau = D_r^{-1}$ denoting the persistence time and $\ell = V_0\tau$ the persistence length. The former corresponds to the typical time it takes for the direction of motion of a tip to become uncorrelated, providing a natural time scale for the growth of the spatial branching pattern. Varying the time scale τ and branching rate R_{branch} accordingly results in networks with identical topology.

During a branching event, two new tips were generated. The parent duct and the two offspring branches were considered to be co-planar, while the angle of rotation or *roll* of this plane around the axis formed by the parent duct was estimated empirically (see Supplementary Figs. 6a-6e), as was the angle formed between the two offspring branches (see below and Supplementary Fig. 6d).

Exponential domain growth was implemented as follows: at each time step, the positions $\mathbf{r}_i(t)$ of every particle (tip or duct) in the system, were scaled by a factor $(1 + R_{\text{exp}}\Delta t)$, which corresponds to a long-term exponential growth of the original domain size in all three dimensions by a factor $\omega(t) = \exp(R_{\text{exp}}t)$, with expansion rate R_{exp}. To account for reactivation of tips, we kept track of all delayed tips in the system and checked, on every iteration and after the expansion of the domain, whether they were still within the inhibition radius of a duct "particle". If this was not the case, the tip was reactivated, allowing it to undergo branching and elongation events. For every realization of the process, we recorded the positions of all particles, their identity and connectivity in the form of an *edge list*. This allowed the reconstruction of the branching tree to study their spatial and topological properties and compare them with the experimental observations.

A. Initialization, boundaries and tip termination criteria

Depending on the context, stochastic simulations were initialized either by placing a single duct at the origin of the expanding domain or by seeding the system with one of the measured ductal tree configurations at E14.5. In the case of the former, the initial duct had only one active tip, with the other end considered as the root of the branching tree. In the latter, statistical distributions were obtained from the average of many stochastic simulations with 1/3 seeded with the measured E14.5 configuration of sample 1, 1/3 from sample 2 and 1/3 from sample 3 (see Supplementary Fig. 3). In all cases, at the initial time-point, all tips were taken as active except for the root of the tree. Stochastic simulations were carried out in an unconfined (open) domain, which allowed tips to propagate outwards without boundary constraint until the maximum simulation time $t_{\mathtt{max}}$ was reached. This choice was consistent with the hypothesis that, during embryonic development, the salivary gland expands and branches freely until there is a global transition to a phase of acini formation, commencing after E16.5, that terminates fully the branching process [2].

B. Estimation of model parameters from the experimental data

As discussed earlier, the persistence time ($\tau = 1/D_r$) provides a time scale for the growth of the branching pattern. With this in mind, the IBDRW has only three key parameters: the ratio of branching rate to rotational diffusion $r_{\text{branch}} = R_{\text{branch}}/D_r$, the inhibition radius ρ_{a}, and the ratio of the gland expansion rate to rotational diffusion $r_{\text{exp}} = R_{\text{exp}}/D_r$. Before estimating the rates, we first extracted the characteristic length and time scale of the tip growth process by estimating the level of persistence of ductal elongation. For this, we measured the duct *tortuosity* [3] at E14.5. Given the duct length L_{d} between two branching points, and the Euclidean distance ℓ_{d} between the two, we defined the duct tortuosity as the relative deviation $\Delta = (L_{\text{d}} - \ell_{\text{d}})/L_{\text{d}} \approx 0.11$. This implied that, on average, ducts were 11% longer than the Euclidean distance between consecutive branching points. For small deviations, the value of the rotational diffusion constant D_r was approximated by Δ, so that the factor $\sqrt{2D_r} \approx \pi/7$ for the salivary gland, with a characteristic time scale of persistence $\tau = 9.1$ h.

To estimate r_{branch}, we first measured the average level reached by each endbud in E14.5 and E18.5 trees. Sampled across $n = 3$ replicates at E14.5, endbuds were, on average, positioned at level 9 ± 1 (average±s.d.), while at E18.5 this increased to level 14 ± 1 (average±s.d.), resulting in a typical difference of about 5 ± 2 (average±s.d.) generations of branching between the two time-points. This indicated that a typical active endbud at E14.5 undergoes around 5

subsequent branching events in the course of 4 days. This yielded a tip branching rate of around $R_{\text{branch}} = 0.05 \pm 0.02$ h^{-1} (average±s.d.) or 1 tip bifurcation event every 19 hours. Rescaling by τ^{-1}, we therefore have $r_{\text{branch}} = 0.45$.

We then proceeded to estimate the duct elongation speed, V_0. For this, we considered the average duct length between consecutive bifurcation events at generations 15 or above. This choice was made to minimize the effect of tissue and duct expansion, which would otherwise cause an effective reduction of the estimate. This resulted in an average duct length of $20 \pm 2\mu$m (average±s.d.), or taking into account the time between branching events, an elongation speed of $V_0 = 1\mu$m/h.

An estimate of the expansion rate R_{exp} was made by measuring the typical diameter of the SMG at different time points (Fig. 2). By fitting exponential growth curves, proportional to $\exp(R_{\text{exp}}t)$, we found that the growth rate R_{exp} for the whole SMG was given by $R_{\text{exp}} \approx 0.014$ h^{-1}, 95% CI $[0.011, 0.017]$ ($n = 3$ repeats for each time point). When focused on the first lobe, we found a reduced rate of $R_{\text{exp}} \approx 0.0135$ h^{-1}, 95% CI $[0.0066, 0.020]$. From this, we considered the expansion for the SMG to be $R_{\text{exp}} \approx 0.01$ h^{-1}, or rescaling by τ^{-1}, $r_{\text{exp}} \approx 0.09$.

To determine the inhibition radius, a rough estimate was made based on the distance between endbuds and the nearest duct, in a conic section in their direction of propagation. This provided an estimated value of $\rho_{\text{a}} \approx 60 \pm 34$ microns (average±s.d.). An alternative estimate, based on the nearest-neighbour distance between acini at the E18.5 time point, resulted in the value $\rho_{\text{a}} \approx 36 \pm 26$ μm (average±s.d.). Note that, in both cases, the range of values of the inhibition radius are broad. This is because its estimate is subject to sources of technical variability. In particular, measurements of tip-to-duct and tip-to-tip distances were carried out by measuring the distance from the center of a duct to the center of an acinus, as this is the quantity that enters in the agent-based simulations. However, the actual size of a duct and acinus may vary from duct to duct, which will affect the estimate of the range of inhibition. As such, the inhibition radius of the model may not translate precisely to the measured estimate. Moreover, within the IBDRW framework, we assumed that tip delay responds only to tip-duct interaction, i.e., volume exclusion. However, in the context of the real biological system, we note that there might be biochemical cues and more complex interactions that contribute to an effective inhibition radius. With these caveats in mind, based on the estimates above, we considered an rough estimate of the inhibition radius of around 50 microns, a value that was then used as a guide to define the reasonable fitting range in the stochastic simulations.

The total developmental time (which translates to the maximum simulation time) was set to 84 hours, or 3.5 days. This assumes that differentiation of tip cells commences at around E18, so that branching is reduced and by E18.5 all endbuds have differentiated into acini. Larger values of the developmental time resulted in larger and more persistent subtrees (see Supplementary Fig. 9e).

The angles formed by offspring branches during a tip bifurcation were estimated from the empirical data. The (periodic) roll angle φ of the plane formed by the two offspring branches followed a peaked distribution that we approximated by a Laplace distribution

$$f(\varphi|\mu, b) = \frac{1}{2b} \exp\left(-\frac{|\varphi - \mu|}{b}\right) \tag{S3}$$

with mean $\mu = 90°$ and scale parameter $b = 21°$ (Figs. 6a-6e). Empirically, the angle formed by two offspring branches at E15.5 was well-fit by a truncated normal distribution, with mean 90° and standard deviation 20°, which increased slightly at E18.5 (Supplementary Fig. 6d). Small changes to these parameters had no significant effect on the quantitative properties of the networks. However, as observed in other contexts [3, 4], large angular deviations do affect the organization, mainly because small branching angles may lead to immediate termination of one or both offspring tips. The quantitative properties remained, to a large extent, unaffected as long as the branching angle and sensing cone angles did not cause immediate (and non-physical) termination events after branching.

To find a best fit of the IBDRW to the experimental data, we generated stochastic simulations of the model for the parameter values defined above, taking as an initial condition one of the three measured rudimentary trees at E14.5. The inhibition radius was varied in the range $\rho_{\text{a}} \in [30, 70]\mu$m and the expansion rate in the range $r_{\text{exp}} \in [0.003, 0.014]$. For each set of parameters, we computed the endbud fraction q_i and distributions of subtree size and persistence from $N = 10^3$ realizations. By comparing the results of stochastic simulations with measurements from the experimental data using an R^2 goodness-of-fit estimate (see the following section), we found the parameter values of r_{exp} and ρ_{a} that provided the best fit to the data. The summary of parameters are shown in Table S1. Alongside fits to the full branching statistics, further predictions were made of the variation in the average duct length with branch level, the duct length distribution and density fluctuations, and the results compared with experiment.

To test whether salivary gland development was consistent with a conventional BARW model, we considered the same parameter estimations defined above for r_{branch} and ρ_{a}, supplemented by the branching angles and developmental time, and considering the ductal networks at E14.5 as the initial conditions of the numerical simulations (see Table S1). However, in this case, we set $r_{\text{exp}} = 0$. As noted in the main text, this model failed to recapitulate the spatial and topological properties of the true ductal networks (Supplementary Fig. 7). In particular, this model predicted much

smaller subtrees, and shorter duct lengths compared to the E18.5 data.

Finally, as emphasized in the main text, the inability of the conventional BARW to predict the growth dynamics of the salivary gland was not surprising. The conventional BARW model predicts two essential features: a linear-like expansion of the network and the self-organization of tip growth to the boundary. By contrast, the salivary gland shows an exponential-like growth characteristic, with proliferatively active tips observed throughout the network during development.

C. Goodness of fit

Fits of the stochastic simulations of the IBDRW model to the experimental data were performed using the coefficient of determination R^2. Given a set of n points to fit in the data $(x_1, x_2, ..., x_n)$, whose average value is given by $\bar{x}$, and a corresponding set of theoretical predictions $(y_1, y_2, ..., y_n)$, and defining $S_{\rm res} = \sum_{i=1}^{n}(x_i - y_i)^2$ and $S_{\rm tot} = \sum_{i=1}^{n}(x_i - \bar{x})^2$, R^2 is defined by

$$R^2 = 1 - \frac{S_{\rm res}}{S_{\rm tot}}. \tag{S4}$$

R^2 takes the value of unity if the prediction fits the data perfectly. In light of the exponential-like decay of many of our observations, such as the duct length distributions (Fig. 2a) and subtree statistics, we computed $R^2_{\rm log}$, defined as the coefficient of determination with the log-transformed inputs x_i and y_i. This gives more weight to those values in the tails of the data, thus providing a better measure of the goodness-of-fit.

D. Sensitivity analysis

To test how strongly the model predictions are influenced by changes to each of the input parameters, we ran stochastic simulations of the full stochastic IBDRW model, choosing a range of parameter values above and below those reference values shown in Table S1, based on direct measurements or fits. The results of this sensitivity analysis (Supplementary Fig. 9) showed that all tested parameters, with the exception of the duct rotational diffusion D_r, had an important effect on the subtree sizes distribution. Variations of the branching rate $R_{\rm branch}$ had a noticeable effect on all statistics (Supplementary Fig. 9a), while variations of the expansion rate had the most effect on the duct length distribution (Supplementary Fig. 9b). Variations in the simulation time (Supplementary Fig. 9d) led to changes in the overall tree sizes and persistence. Similarly to the branching rate, variations of the annihilation radius also affected all statistics. On the other hand, changes to the rotational diffusion had little effect on the spatial and topological measures (Supplementary Fig. 9e).

E. Zero-dimensional stochastic branching and termination model

As an alternative hypothesis to explore the branching dynamics, we considered a zero-dimensional model in which the chance of branching and termination was assumed to be autonomous, regulated at the level of individual tips, and independent of spatial location within the tissue and local duct density [5, 6]. In this scenario, the statistical organization of the salivary gland branching tree is characterised fully by the probability q_i that a tip terminates at a given level, here referred to as *endbud fraction*. Mathematically, this model corresponds to a stochastic birth-death (BD) type process, where the branching probability depends on the generation in the tree. To test this model, we implemented numerically a discrete model in which particles a, representing active tips, were allowed to branch $a \longrightarrow a + a$ with probability $1 - q_i$ or become inactive (s), $a \longrightarrow s$ with probability q_i, where q_i depends on the level i and was extracted empirically from the experimental data at E18.5, as depicted in Supplementary Fig. 7a.

Numerically, stochastic simulations were performed by initialising the system with a single particle at level 1, and on every iteration an active tip, chosen at random, was allowed to branch or terminate with a probability that depended only on its branch level. Simulations terminated once all tips in the tree became inactive. We compared the empirical observations with the predictions of the model from $N = 10^3$ realizations (see BD in Figs. 7a and 7d). These results show that a simple birth-death model fails to recapitulate the statistical properties of the salivary gland branching tree, predicting drastically smaller but more persistent trees than found experimentally. These observations suggest that spatial, extrinsic, constraints are key regulators of the topological organization of the salivary gland epithelium.

S2. MEAN-FIELD THEORY

The agent-based implementation of the IBDRW model allowed us to quantify the statistical properties and dynamics of the model and compare the results with experimental measurements from the developing salivary gland network. However, such an implementation does not provide analytical insights into the dynamics of the growth process, nor can it identify which parameter combinations are key to the dynamics. To gain further insight into the dynamics of the IBDRW, we turned to an effective hydrodynamic or mean-field theory of the stochastic branching process. The mean-field theory can be motivated as a coarse-grained description of the dynamics or, more formally, obtained as the minimum action of the non-equilibrium field theory of the stochastic IBDRW process [7]. Although, the mean-field approximation is not capable of predicting the statistical organization of the network, it can define how the local average density of active and inactive endbuds, as well as ducts, evolve as a function of developmental time.

A. Mean-field theory of the BARW model

To develop the mean-field theory of the IBDRW model, we start by first defining the set of "reactions" for the composite components of the network in the conventional BARW. Formally, active tips (a) are modeled as point-like particles that undergo a persistent random walk. As an active particle moves, it leaves behind a trail of inactive ducts (i), that constitute the ductal network. When an active tip becomes within an inhibition radius of an active tip or duct, it becomes inactive. This process can be represented as the set of local reactions [3]

$$a \overset{R_{\text{branch}}}{\longrightarrow} a + a \tag{S5a}$$

$$a \overset{R_{\text{duct}}}{\longrightarrow} a + i \tag{S5b}$$

$$a + i \overset{R_{\text{delay}}}{\longrightarrow} i + i \tag{S5c}$$

$$a + a \overset{R_{\text{delay}}}{\longrightarrow} i \tag{S5d}$$

where R_{branch}, R_{duct}, and R_{delay} correspond to the branching, duct creation, and inhibition rates, respectively. In this framework, the inhibition radius need not be incorporated explicitly. Instead, we consider that all reactions occur locally, and that the effect of the annihilation radius is subsumed into the effective inhibition rate, R_{delay}. Here, we consider only dichotomous branching in which an active tip branches into precisely two new active tips. This could be generalised by considering a distribution in the number of offspring, $P(n)$, with $n \in \mathbb{N}$, so that $a \longrightarrow na$. However, it has been shown that the long-term dynamics of such systems is always dominated by dichotomous branching [8].

Approximating the motion of active particles by a diffusive process with diffusion constant D, the set of reactions (S5) may be approximated, at the mean-field level, by a set of partial differential equations (PDEs) describing the spatio-temporal evolution of the space- and time-dependent densities, $A(\mathbf{x}, t) = [a]$ and $I(\mathbf{x}, t) = [i]$, of active and inactive (i.e., duct) particles [3], respectively:

$$\frac{\partial A}{\partial t} = D\nabla^2 A + R_{\text{branch}} A - R_{\text{delay}} A(A + I) \tag{S6a}$$

$$\frac{\partial I}{\partial t} = R_{\text{duct}} A + R_{\text{delay}} A(A + I). \tag{S6b}$$

In the framework of the BARW model (S6), tip-driven growth is constrained by steric effects on a fixed domain, so that temporal changes in the ductal network occur only in response to the activity of tips. Active tips submerged within the trailing ductal network become rapidly terminated so that growth becomes driven by proliferation of active tips that self-organize as a soliton pulse at the expanding front of the epithelial network. As this pulse propagates, it leaves in its wake a spatially uniform distribution of inactive ductal tissue. As noted in the main text, more complex dynamics, such as a persistent random walk (or active Brownian motion), may be introduced at the mean-field level [9]. However, as these refinements enter as gradient terms, they would not effect the long-term steady-state dynamics of the expanding network.

B. Mean-field theory of the IBDRW

In the IBDRW model, branching of the ductal epithelium takes place on the background of an expanding domain (Figs. 1 and 2f). The expansion of the domain has three effects on network development: 1) the promotion of a

constant linear-expansion of ducts to match precisely the expansion of the tissue and preserve the connectivity of the network; and 2) a local decrease of epithelial density, which leads to 3) the reactivation of delayed tips due the increase in available space. The second effect is accommodated at the mean-field level when considering the dynamics on an expanding domain. Persistent duct expansion and tip reactivation may be incorporated directly into the reaction-diffusion scheme by introducing a third particle species s corresponding to delayed tips that are generated upon inhibition of active tips and reactivated at rate constant R_{react}. In this case, the set of reactions for the IBDRW model takes the form

$$a \xrightarrow{R_{\text{branch}}} a + a \qquad \text{tip branching} \tag{S7a}$$

$$a \xrightarrow{R_{\text{duct}}} a + i \qquad \text{duct creation} \tag{S7b}$$

$$i \xrightarrow{R_{\text{elong}}} i + i \qquad \text{duct expansion} \tag{S7c}$$

$$a + i \xrightarrow{R_{\text{delay}}} s + i \qquad \text{tip delay} \tag{S7d}$$

$$a + a \xrightarrow{R_{\text{delay}}} s \qquad \text{tip delay} \tag{S7e}$$

$$a + s \xrightarrow{R_{\text{delay}}} s + s \qquad \text{tip delay} \tag{S7f}$$

$$s \xrightarrow{R_{\text{react}}} a \qquad \text{tip reactivation.} \tag{S7g}$$

The fact that ducts co-expand with the domain means that the duct expansion rate R_{elong} coincides precisely with the linear expansion rate of the system, i.e., $R_{\text{elong}} = R_{\text{exp}}$. Moreover, since the reactivation of endbuds follows directly from the expansion of tissue as new space is opened, we may set $R_{\text{react}} = R_{\text{exp}}$.

Before incorporating the background expansion, at the mean-field level, the set of reactions (S7) may be cast as a set of kinetic equations for the densities of active tips $A(x,t) = [a]$, inactive ducts $I(x,t) = [i]$ and delayed tips $S(x,t) = [s]$,

$$\frac{\partial A}{\partial t} = D\nabla^2 A + R_{\text{branch}}A - R_{\text{delay}}A(A + S + I) + R_{\text{exp}}S \tag{S8a}$$

$$\frac{\partial S}{\partial t} = R_{\text{delay}}A(A + S + I) - R_{\text{exp}}S \tag{S8b}$$

$$\frac{\partial I}{\partial t} = R_{\text{exp}}I + R_{\text{duct}}A, \tag{S8c}$$

with the no-flux boundary condition ($\nabla A = 0$, etc.). Here, the duct expansion rate, R_{elong}, and tip reactivation rate, R_{react}, have been replaced by the system expansion rate R_{exp}. As a consistency check, we can see that, in the non-expanding system ($R_{\text{exp}} = 0$), we recover Eq. (S6) by considering delayed tips as belonging to the inactive compartment, i.e., $S \longrightarrow I$.

To proceed, we now consider the influence of the domain expansion on the mean-field equations (S8). Following Crampin et al. [10], where the effect of domain growth was investigated in the context of spatial pattern formation, we considered a general particle density field $C(\mathbf{x}, t)$, whose dynamics is governed by a reaction-diffusion equation of the form

$$\frac{\partial C}{\partial t} = D\nabla^2 C + \mathcal{R}(C) \tag{S9}$$

with $\mathcal{R}(C)$ representing the local reaction terms. A domain Ω_t, whose topology may depend on time t, introduces a flow field $\mathbf{u}(\mathbf{x}, t)$, whose effects are captured by a term of the form $\nabla \cdot (\mathbf{u}C)$. In this case, the dynamics of the density field C on an expanding domain is governed by the equation [10]

$$\frac{\partial C}{\partial t} + \nabla \cdot (\mathbf{u}C) = D\nabla^2 C + \mathcal{R}(C). \tag{S10}$$

Applied to Eqs. (S8), this leads to the mean-field equations

$$\frac{\partial A}{\partial t} + \nabla \cdot (\mathbf{u}A) = D\nabla^2 A + R_{\text{branch}} A - R_{\text{delay}} A(A + I + S) + R_{\text{exp}} S \tag{S11a}$$

$$\frac{\partial S}{\partial t} + \nabla \cdot (\mathbf{u}S) = R_{\text{delay}} A(A + I + S) - R_{\text{exp}} S \tag{S11b}$$

$$\frac{\partial I}{\partial t} + \nabla \cdot (\mathbf{u}I) = R_{\text{exp}} I + R_{\text{duct}} A. \tag{S11c}$$

The additional terms introduce two new contributions to each field equation: i) advection terms of the form $\mathbf{u} \cdot \nabla C$, and ii) a dilution term of the form $C(\nabla \cdot \mathbf{u})$, which when expanded leads to Eq. (1) of the main text.

Experimental observations suggest that the mesenchymal tissue undergoes sustained and spatially uniform proliferation, consistent with the observed exponential-like expansion of the developing gland. Here, we assume that growth occurs uniformly across the tissue so that the expansion is affine, preserving locally geometric properties and thus duct morphology. At the same time, we assume that epithelial cells proliferate to preserve the continuity of ducts and branch angles. With this in mind, we may now describe the flow $\mathbf{u}$ caused by the expanding domain Ω_t as a temporal evolution of the spatial coordinates $\mathbf{x}(t)$, so that $\mathbf{u} = \frac{\mathrm{d}\mathbf{x}}{\mathrm{d}t}$. Considering a uniform, isotropic, domain expansion, we can relate $\mathbf{x}(t)$ at time t, with the coordinates $\mathbf{y} = \mathbf{x}(0)$ of the original domain Ω_0 by a space-independent function $\omega(t)$ [11]

$$\mathbf{x}(t) = \omega(t)\mathbf{y} \tag{S12}$$

where $\omega(t = 0) = 1$, such that

$$\mathbf{u}(\mathbf{x}, t) = \frac{\mathrm{d}\omega(t)}{\mathrm{d}t}\mathbf{y}. \tag{S13}$$

For an exponential expansion of the domain, $\omega(t) \propto \exp(R_{\text{exp}} t)$, with rate constant R_{exp}, we can write the divergence of the flow, or *dilution factor*, as [11, 12]

$$h(t) = \nabla \cdot \mathbf{u} = d\frac{\dot{\omega}(t)}{\omega(t)} = R_{\text{exp}} d \tag{S14}$$

for spatial dimensions $d \geq 1$, where $R_{\text{exp}} d$ defines the *volumetric* expansion rate. Note that only for an exponential expansion at constant rate is the ratio $\dot{\omega}(t)/\omega(t)$ time-independent. Using Eq. (S14), rescaling the rates by the duct creation rate R_{duct} and time by R_{duct}^{-1}, space by $(D/R_{\text{duct}})^{1/2}$, defining the dimensionless flow field $\mathbf{v} = \mathbf{u}/(R_{\text{duct}} D)^{1/2}$, and introducing the rescaled density fields $a = AR_{\text{delay}}/R_{\text{branch}}$ (similarly for S and I), the kinetic equations (1) are written in the dimensionless form as

$$\frac{\partial a}{\partial t} + \mathbf{v} \cdot \nabla a + r_{\text{exp}} da = \nabla^2 a + r_{\text{branch}} a(1 - (a + i + s)) + r_{\text{exp}} s \tag{S15a}$$

$$\frac{\partial s}{\partial t} + \mathbf{v} \cdot \nabla s + r_{\text{exp}} ds = r_{\text{branch}} a(a + i + s) - r_{\text{exp}} s \tag{S15b}$$

$$\frac{\partial i}{\partial t} + \mathbf{v} \cdot \nabla i + r_{\text{exp}} di = a + r_{\text{exp}} i. \tag{S15c}$$

From this result, the effect of the spatial dimension of the system is made clear: as ducts (I) always co-expand with the system, their dilution occurs at a lower rate than that of active (A) and delayed (S) tips. Note that, in dimension $d = 1$, ducts (I) undergo no dilution at all since, in this case, the ducts and the system expand identically.

1. *Transformation of the mean-field equations to a co-moving frame*

Based on this result, we can now represent the reaction-diffusion system (S11) on an isotropically growing domain as a system with time-dependant parameters on a stationary domain [10]. Expressing the active tip density field in terms of the expanding variables $a(\mathbf{x}, t) = a(\omega(t)\mathbf{y}, t)$, and equivalently for the i and s fields, with $\omega = \exp(r_{\text{exp}} t)$,

Eqs. (S11) may be written as

$$\frac{\partial a}{\partial t} + r_{\rm exp} d a = \frac{1}{\omega^2(t)} \nabla^2 a + r_{\rm branch} a(1 - (a + i + s)) + r_{\rm exp} s \tag{S16a}$$

$$\frac{\partial s}{\partial t} + r_{\rm exp}(d+1) s = r_{\rm branch} A(a + i + s) \tag{S16b}$$

$$\frac{\partial i}{\partial t} + r_{\rm exp}(d-1) i = a, \tag{S16c}$$

where the advection term is eliminated in favor of a time-dependent effective diffusion constant that decays as $1/\omega^2(t) = \exp(-2 r_{\rm exp} t)$.

2. Homogeneous solutions

The system of equations (S16) has two homogeneous steady-state solutions: an empty state $\rho_0 = (a_0, s_0, i_0) = (0, 0, 0)$, and an active state $\rho_+ = (a_+, s_+, i_+)$ that exists for $d > 1$, where

$$a_+ = \frac{r_{\rm exp}(d^2-1)(r_{\rm branch} - r_{\rm exp} d)}{r_{\rm branch}(r_{\rm branch}(d-1) + d)} \tag{S17a}$$

$$s_+ = \frac{(r_{\rm branch} - r_{\rm exp} d)}{r_{\rm exp} d} a_+ = \frac{(d^2-1)(r_{\rm branch} - r_{\rm exp} d)^2}{d r_{\rm branch}(r_{\rm branch}(d-1) + d)} \tag{S17b}$$

$$i_+ = \frac{1}{r_{\rm exp}(d-1)} a_+ = \frac{(d+1)(r_{\rm branch} - r_{\rm exp} d)}{r_{\rm branch}(r_{\rm branch}(d-1) + d)}. \tag{S17c}$$

This solution is stable when the branching rate is larger than the volumetric expansion rate, i.e., $r_{\rm branch} \geq d r_{\rm exp}$ (or, equivalently, $R_{\rm branch} \geq d R_{\rm exp}$), and both branching and expansion rates remain non-zero. For the non-expanding case, the only homogeneous steady-state is the empty state ρ_0, as obtained in the standard BARW model [3]. In Eq. (S16), the empty state ρ_0 becomes unstable when $r_{\rm branch} > d r_{\rm exp}$, such that any (positive) perturbation away from ρ_0 grows toward the positive homogeneous steady state ρ_+. Fronts connecting both homogeneous solutions, ρ_0 and ρ_+, may then propagate onto the unstable state, leaving in its wake uniform densities of ducts, and active and inactive tips. The solutions (S17) imply that, for a given dimension $d > 1$, the ratio of active tips to ducts $i_+/a_+ = r_{\rm exp}(d-1)$ is controlled by the expansion to duct creation rate ratio $r_{\rm exp}$, while the ratio of ducts to overall tip density $i_+/(a_+ + s_+) = d/(r_{\rm branch}(d-1))$ is inversely proportional to the branching to duct creation rate ratio $r_{\rm branch}$.

C. Numerical integration of the mean-field equations

The numerical solution of the mean-field equations (S16) was obtained using a finite difference scheme and a 9-point stencil for the discrete approximation of the Laplacian (diffusion) operator. For the numerical results shown in Fig. 4l, we considered a lattice spacing $dx = 0.1$, a time-discretization $dt = 0.01$, measured in units of $(R_{\rm duct}/D)^{1/2}$ and $1/R_{\rm duct}$, respectively, a system size of $L = 200$ points, and dimensionless rates $r_{\rm branch} = 1$ and $r_{\rm exp} = r_{\rm branch}/8$. As mentioned in the main text, the mean-field rates do not have a trivial correspondence to the microscopic rates, yet we do expect that the relative ratio $r_{\rm branch}/r_{\rm exp} \approx 8$ to be conserved. We considered no-flux boundary conditions at $x = 0$ and $x = L$, and periodic boundaries elsewhere, and the system was initialized with a localized perturbation at $x = 0$ on the active particle field, i.e., $a(x, t = 0) = \delta(x)$ and $i(x, t = 0) = s(x, t = 0) = 0$. In the regimen considered ($r_{\rm branch} > r_{\rm exp} d$), the active state ρ_+ given by Eqs. (S17) is stable so that, once initialized as described above, the system converges rapidly toward that state. The front connecting the stable (ρ_+) and unstable (ρ_0) states exhibits a peak of density above ρ_+ that decays on the back of the front with a damped oscillatory profile (Fig. 4l). Once the front has passed, the system reaches a stable stationary state, supported by a constant rate of tip branching and duct creation, which compensate precisely the diluting effect of the expanding domain.

S3. BRANCHING STATISTICS OF INDEPENDENT LOBES OF THE SMG

To explore the dynamics of branching in the salivary gland, we focused our analysis on the first lobe of the SMG, one of its largest components. To explore branching statistics on different lobes, we took advantage of results from an independent investigation of the role of Kras signalling on lineage specification during salivary gland development (see Data and Code Availability). Here, we made use of an inducible genetic labelling system – the Red2Kras confetti mouse model [13] – to study at clonal density the interaction of RFP+ clones active for Kras with wildtype clones carrying a different confetti color. Glands were induced at clonal induction frequency at E13.5, collected and imaged, and their networks manually segmented at E18.5. Clones mutant for Kras showed evidence of a dynamics different from that of wildtype confetti clones. However, at low (clonal) levels of induction, there was no evidence for large-scale changes in the branching characteristics of glands due to the minority of mutant clones. Notably, comparison of the branching statistics of the E18.5 control (confetti) samples with two Kras E18.4 samples from lobes 2 and 4 of the SMG showed that the latter two lobes fell broadly within the range of inter-sample variability of the confetti system (Supplementary Fig. 12). The fact that different lobes follow the same trends as the confetti data suggests a robust and conserved pattern of branching morphogenesis across the gland, with the IBDRW model describing the large-scale organisation of the salivary gland networks.

S4. BRANCHING DYNAMICS IN OTHER BRANCHING CONTEXTS

Previous studies of branching dynamics of the mouse mammary gland and kidney ductal epithelium suggested that the large-scale organisation of both tissues were predicted well by the standard (non-inflationary) BARW model [3]. In the case of the mammary gland, branching morphogenesis takes place within a preformed mesenchymal tissue known as the fat pad. In this case, expansion is driven by tip-localized progenitors and, as cells leave the tip, they undergo limited proliferation before exiting cell cycle, giving rise to a simple stratified epithelium. In this case, ducts remain largely non-proliferative during the postnatal phase of development. By contrast, in the case of kidney, the embryonic development of the ductal epithelium does not take place in the context of an inactive preformed mesenchyme, but both populations expand cooperatively during development. Moreover, proliferation kinetics measurements show that neither ductal cells nor tip cells in “submerged” endbuds (i.e., endbuds that are positioned away from the periphery of the growing gland) fall out of cycle. It therefore makes sense to question why the statistics of the branched network can be captured by the BARW paradigm.

To address this question, it is helpful to note that the original BARW model is not altogether distinct from the inflationary BARW model. It is instead a limit of the latter model in which the mesenchmye does not contribute to the local expansion of the ducts. In this limit, where the growth rate vanishes, even if submerged tips would have the capacity to continue branching, since there is no local expansion of the surrounding tissue, steric constraints are never alleviated. But what happens when glands “inflate” during the branching process, but the inflation rate is small? Here, it is necessary to question the meaning of “small”. Without inflation, the expansion of the ductal network is driven by active tips that become localized at the periphery of the branching network. Tips that become submerged within the network become rapidly inhibited by steric constraints. In this limit, the radial extent of the network expands linearly in time, as predicted by the mean-field dynamics and illustrated in the stochastic simulations of the BARW model. Indeed, such a linear growth characteristic has been demonstrated in the context of the mouse mammary gland development alongside the restriction of proliferation to endbuds at the periphery of the ductal network.

What happens when the inflation rate is non-vanishing? If the expansion of the mesenchymal tissue provides an additional linear-like radial growth dependence, we would expect some contribution from the reactivation of submerged tips as steric constraints become alleviated by the expansion. However, in the long-term limit, such contributions have only a marginal influence on the statistics of branching since such branching events would be infrequent and dwarfed by primary contributions from branching at the periphery of the growing ductal network. By contrast, when the inflation of the mesenchymal tissue leads to a radial expansion that increases exponentially in time, in the long-term limit, the contribution from submerged tips becomes substantial and competitive with peripheral branching, impacting significantly on the branch statistics. To understand why, it is helpful to consider the likelihood that submerged tips can “catch-up” with the surrounding expanding tissue if the expansion is either linear or exponential. In the long term, when the expansion of the tissue is linear, if a tip propagates at a constant speed relative to the expanding tissue, then no matter how small is the ratio of the expansion and propagation speeds, a submerged tip will always eventually catch up with the expanding network (cf. the well-known problem of an “ant on a rubber rope”), which would necessarily cause a temporal arrest in tip proliferation. By contrast, when the expansion is exponential, in the long-term tips would never fully arrest, but their advance would only be delayed by steric influences. This aligns with the results of the long-term stochastic simulations for the salivary gland. However, one can also question

what happens at short or intermediate time scales, when the branching networks are limited in size. In this case, the dynamics under a linear or exponential growth characteristic will converge – in other words, an exponential expansion begins as a linear-like expansion, viz.

$$\exp[R_{\rm exp}(t_0+t)] \approx \exp[R_{\rm exp}t_0](1+R_{\rm exp}t+O(t^2)) \tag{S18}$$

It takes a characteristic time ($t\ 1/R_{\rm exp}$) before the nonlinearity begins to impact on the growth dynamics. A comparison of the total salivary gland growth (Fig. 3f) is consistent with an exponential-like expansion, which is why we have taken an exponential growth characteristic as a basis to analyse the model. However, for the first lobe of the SMG (Fig. 3f) and also for the kidney (Supplementary Fig. 8a), the expansion does fit well with a linear growth characteristic.

With this in mind, let us consider the results from previous studies of the embryonic development of the mouse kidney. Measurements of the lateral extension of the kidney indicate that, from E11.5 to E19.5 [14], where the dynamics is characterised by branching morphogenesis, the growth characteristic is approximately linear in time (Supplementary Fig. 8a). Although such behavior is consistent with the dynamics of a tip-driven BARW, one cannot conclude that there is no attendant growth of the underlying mesenchymal tissue. On the contrary, there is evidence that the mesenchyme does continues to grow during this phase, and that the submerged tips and ducts continue to proliferate [15].

Therefore, to investigate quantitatively the effects of a linear-like inflation of the mesenchymal tissue on the statistical organization of the branching network, we performed stochastic simulations of the conventional BARW (i.e., without inflation of the surrounding mesenchyme) and compared the resulting branching topologies to those produced by variants of the IBDRW in which the time-dependence of the inflation is either linear or exponential in time (Figs. 8b-8e). These simulations were performed in a three-dimensional setting, taking a single stalk as the initial condition. In all cases, we considered isotropic branching, setting all parameters of the model to be identical but for the inflation characteristics. The expansion rate for the exponential inflation was set to 0.45 (in units of the rotational diffusion constant) and, for the linear growth, the expansion was proportional to time, with proportionality constant equal to 0.45.

The results of these simulations (Figs. 8b-8e) show that, for the chosen parameters, when the inflation is linear in time, the tree statistics are largely indistinguishable from those of the conventional BARW. This is because the network expansion is dominated by peripheral tip branching, as in the BARW model (Supplementary Fig. 8f). More generally, if we would increase the rate of linear gland expansion, there would be a transient phase in which submerged tips contribute significantly to the branching organization. However, in general, based solely on the analysis of branching statistics, it is not possible to discriminate between a BARW and IBDRW with a slow linear-like inflationary expansion of the surrounding mesenchyme. Whether the linear-like expansion of the kidney does indeed allow for a slow persistent reactivation of submerged tips, or chemical influences from maturing ducts and/or surrounding mesenchyme inhibit the resumption of branching of submerged tips, remains a subject for future investigation.

These results show that, in slowly growing branching tissues, the branching dynamics may be well-described within the framework of the non-inflationary BARW. However, if the expansion of the surrounding mesenchymal tissue is super-linear or exponential, the reactivation of submerged tips and remodelling of the ductal epithelium may shape the statistical organization of the branching network in a manner that may conform to the IBDRW model.

TABLE OF PARAMETERS

Parameter	Empirical/Fit	Value for simulations of E16.5/E18.5
R_{branch} (dimension-full)	Empirical	0.05 h^{-1}
r_{branch} (dimension-less)		0.45
R_{exp} (dimension-full)	Best fit	0.006 h^{-1}
r_{exp} (dimension-less)		0.054
Annihilation radius: ρ_{a}	Best fit	45 μm
Initial condition	Empirical	Rudimentary tree at E14.5 (see Supplementary Figs. 3a-3c)
Simulation time	Empirical	48/84 h
Duct tortuosity (Δ)	Empirical	0.11
Branch angle α (Supplementary Figs. 6a-6d)	Empirical	(Normal Dist.) mean: 90° SD: 20°
Branch angle φ (Supplementary Figs. 6a-6e)	Empirical	(Laplace Dist. Eq. (S3)) mean: $\mu = 90°$, scale: $b = 24°$

TABLE S1: Parameters considered in the numerical simulations of the stochastic IBDRW model. The second column indicates whether the parameter used in the numerical simulations was estimated directly from empirical data or fitted, while the third column shows the values used in the simulation of the E16.5 and E18.5 time points. Note that here only the simulation time is different. For simulations of the BARW model the same parameter values were used but the expansion rate r_{exp} was set to zero.

[1] Giorgio Volpe, Sylvain Gigan, and Giovanni Volpe, "Simulation of the active brownian motion of a microswimmer," American Journal of Physics **82**, 659–664 (2014).

[2] Lemonia Chatzeli, Ignacio Bordeu, Seungmin Han, Sara Bisetto, Zahra Waheed, Bon-Kyoung Koo, Maria P Alcolea, and Benjamin D Simons, "A cellular hierarchy of notch and kras signaling controls cell fate specification in the developing mouse salivary gland," Dev. Cell **58**, 94–109 (2023).

[3] E Hannezo, CLGJ Scheele, M Moad, N Drogo, R Heer, RV Sampogna, J Van Rheenen, and BD Simons, "A unifying theory of branching morphogenesis," Cell **171**, 242–255 (2017).

[4] S Shree, S Sutradhar, O Trottier, Y Tu, X Liang, and J Howard, "Dynamic instability of dendrite tips generates the highly branched morphologies of sensory neurons," bioRxiv (2021).

[5] CLGJ Scheele, E Hannezo, MJ Muraro, A Zomer, NSM Langedijk, A Van Oudenaarden, BD Simons, and J Van Rheenen, "Identity and dynamics of mammary stem cells during branching morphogenesis," Nature **542**, 313–317 (2017).

[6] Sebastián Echeverría-Alar, Marcel G Clerc, and Ignacio Bordeu, "Emergence of disordered branching patterns in confined chiral nematic liquid crystals," Proc. Natl. Acad. Sci. U.S.A. **120**, e2221000120 (2023).

[7] JL Cardy and P Grassberger, "Epidemic models and percolation," Journal of Physics A: Mathematical and General **18**, L267–L271 (1985).

[8] I Bordeu, S Amarteifio, R Garcia-Millan, B Walter, N Wei, and G Pruessner, "Volume explored by a branching random walk on general graphs," Sci. Rep. **9**, 1–9 (2019).

[9] Michael E Cates and Julien Tailleur, "When are active brownian particles and run-and-tumble particles equivalent? consequences for motility-induced phase separation," EPL **101**, 20010 (2013).

[10] EJ Crampin, EA Gaffney, and PK Maini, "Reaction and diffusion on growing domains: scenarios for robust pattern formation," Bull. Math. Biol. **61**, 1093–1120 (1999).

[11] A Madzvamuse, EA Gaffney, and PK Maini, "Stability analysis of non-autonomous reaction-diffusion systems: the effects of growing domains," J. Math. Biol. **61**, 133–164 (2010).

[12] G Hetzer, A Madzvamuse, and W Shen, "Characterization of turing diffusion-driven instability on evolving domains," Discrete Contin. Dyn. Syst. **32**, 3975–4000 (2012).

[13] MK Yum, S Han, J Fink, SHS Wu, C Dabrowska, T Trendafilova, R Mustata, L Chatzeli, R Azzarelli, I Pshenichnaya, E Lee, E England, JK Kim, DE Stange, A Philpott, JH Lee, BK Koo, and BD Simons, "Tracing oncogene-driven remodelling of the intestinal stem cell niche," Nature **594**, 442–447 (2021).

[14] Rosemary V Sampogna, Laura Schneider, and Qais Al-Awqati, "Developmental programming of branching morphogenesis in the kidney," J. Am. Soc. Nephrol. **26**, 2414–2422 (2015).

[15] Kieran M Short, Alexander N Combes, Valerie Lisnyak, James G Lefevre, Lynelle K Jones, Melissa H Little, Nicholas A Hamilton, and Ian M Smyth, "Branching morphogenesis in the developing kidney is not impacted by nephron formation or integration," Elife **7**, e38992 (2018).

SUPPLEMENTARY FIGURES

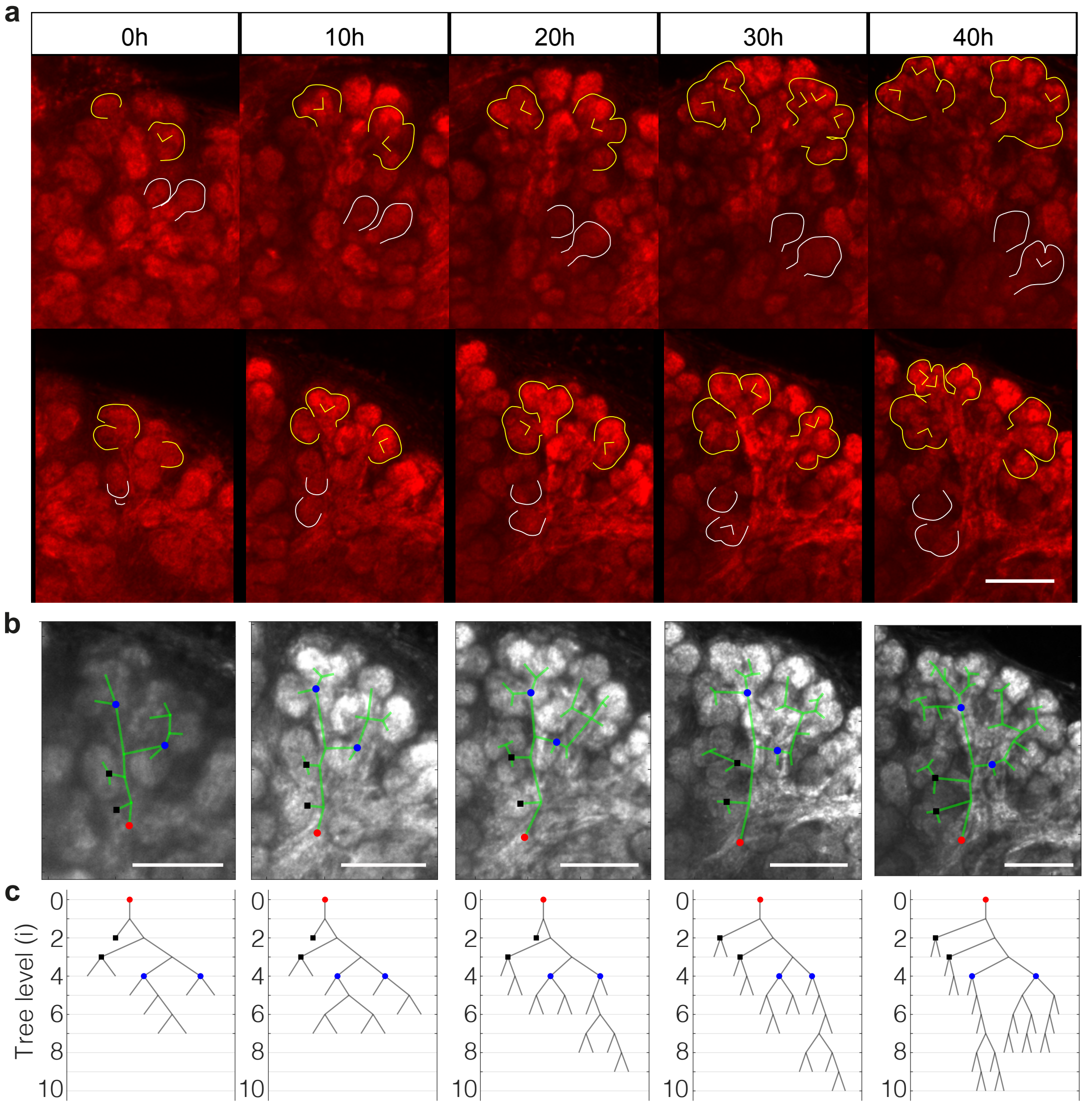

FIG. 1: **Time lapse images of mTmG, E14.5 salivary gland explants cultured for 2 days a**, Stills every 10 hours (h) are shown (see Movie S1, explant 1). Yellow lines outline peripheral endbuds at 0 h. White lines outline submerged endbuds at 0 h. **b** Segmentation of the subtree in the bottom panels in (a) is shown in green. The location of the root of two submerged branches are highlighted in black, roots of branches with access to the peripheral region of the tissue are highlighted in blue. **c** Branching tree representation of the ductal network, with the nodes highlighted as in the top panels. Note that submerged tips are still able to proliferate but undergo only a limited degree of branching over the time window, while endbuds belonging to the peripheral branches maintained a highly branching state. All scale bars correspond to 200μm.

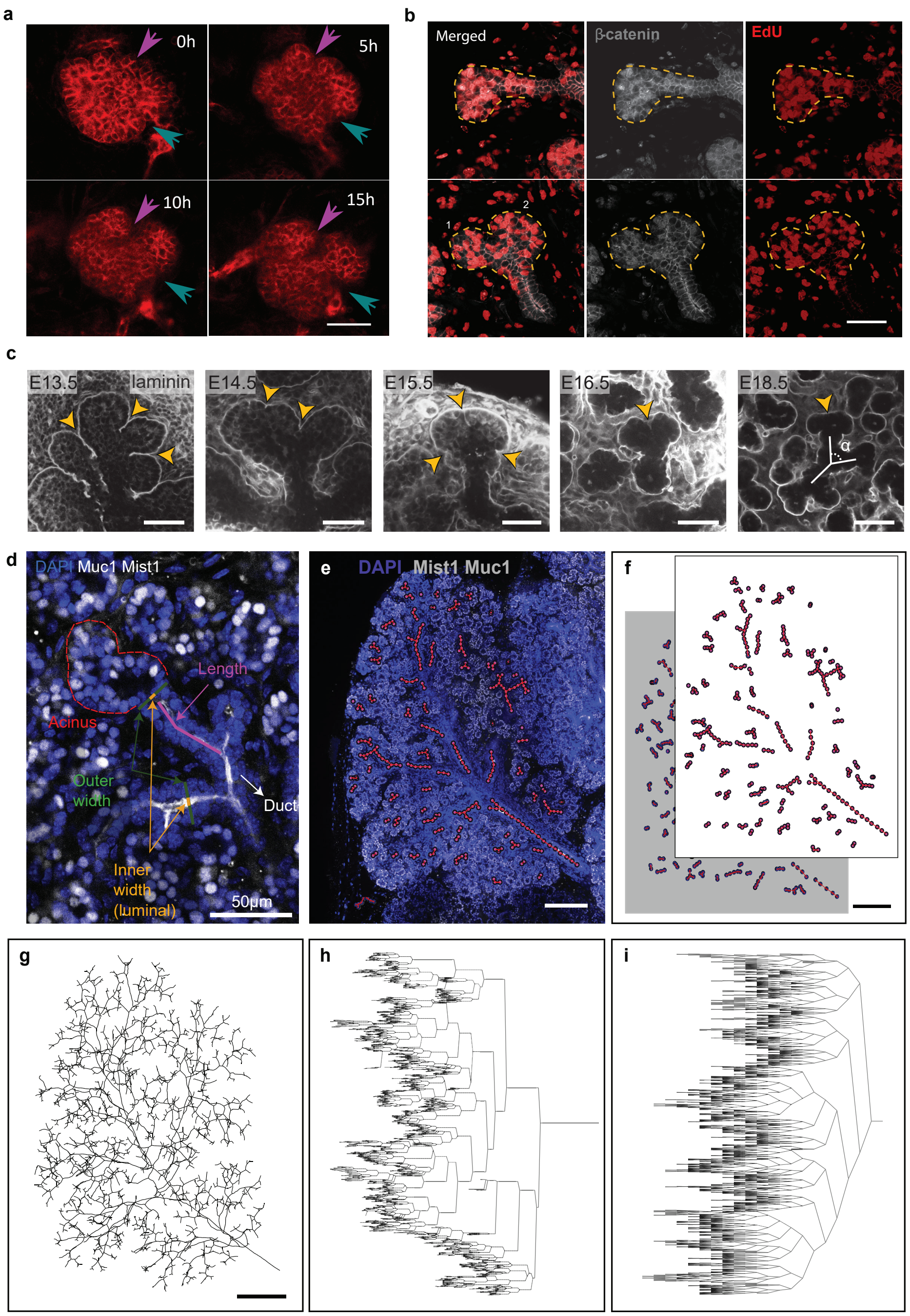

FIG. 2: **Characterization of ductal branching across the developmental time course.** **a**, Time-lapse images of the "clefting" process of an mTmG gland explant at E15.5, from the initiation of two indentations (arrows) at $t = 0$ h, to the branching of the tip at $t = 15$ h. **b**, EdU incorporation in active tips (dashed outline) pre- (upper panels) and post-branching (lower), showing a high proliferative activity of active tips (EdU) and membrane (β-catenin) staining. Scale bars in (a-b) correspond to 60 μm. **c**, Endbuds and clefts in the developing SG at different embryonic time points, as indicated. The epithelium is stained with laminin and clefts are indicated by arrowheads. Scale bars 20 μm. Note that, at the early time points, multiple clefts form simultaneously in a single tip, leading to an early diversification of new endbuds. Later in development, branching events are dichotomous. **d** Schematic of the manual segmentation of the ductal networks and measurement of inner and outer duct widths. **e-i**, Steps for 3D ductal reconstruction. Whole-mount salivary glands were stained with Mist1 (nuclear grey) to identify acini and Muc1 (luminal grey) to stain the lumen of ducts. Nuclei were stained with DAPI (blue). Ducts were manually traced throughout the z-stack (see Methods). Purple dotted line shows the network drawn on top of the ducts. The segmented z-stacks (f) were combined to form a 3D skeleton (g), also represented in (h) in 2d. (i) The branching tree, where each note represents either a branching point or an endbud was constructed from (h). Scale bars in (e-g): 200μm.

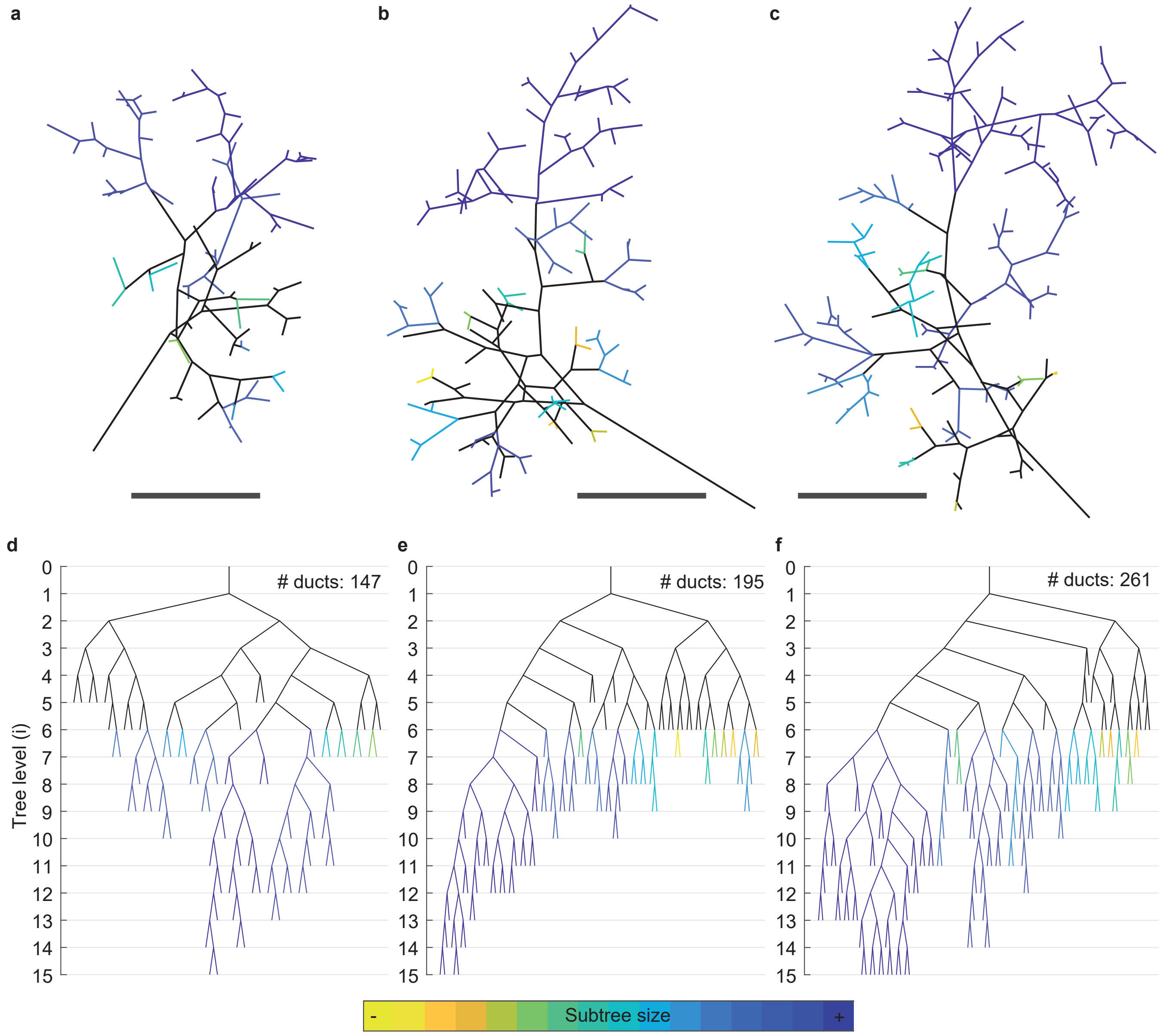

FIG. 3: **Reconstruction of first lobe of the mouse SMG at E14.5. a-c**, XY-projection of the ductal organization of the first lobe of the mouse SMG obtained from segmentation of the 3d confocal images. Individual subtrees from branch level 6 are indicated by different colors. All scale bars correspond to 200μm. **d-f**, Branching trees corresponding to the projections shown in (a-c), indicating the number of ducts in the tree, where subtrees are colored using the same color-code as in (a-c), as indicated by the color bar (bottom).

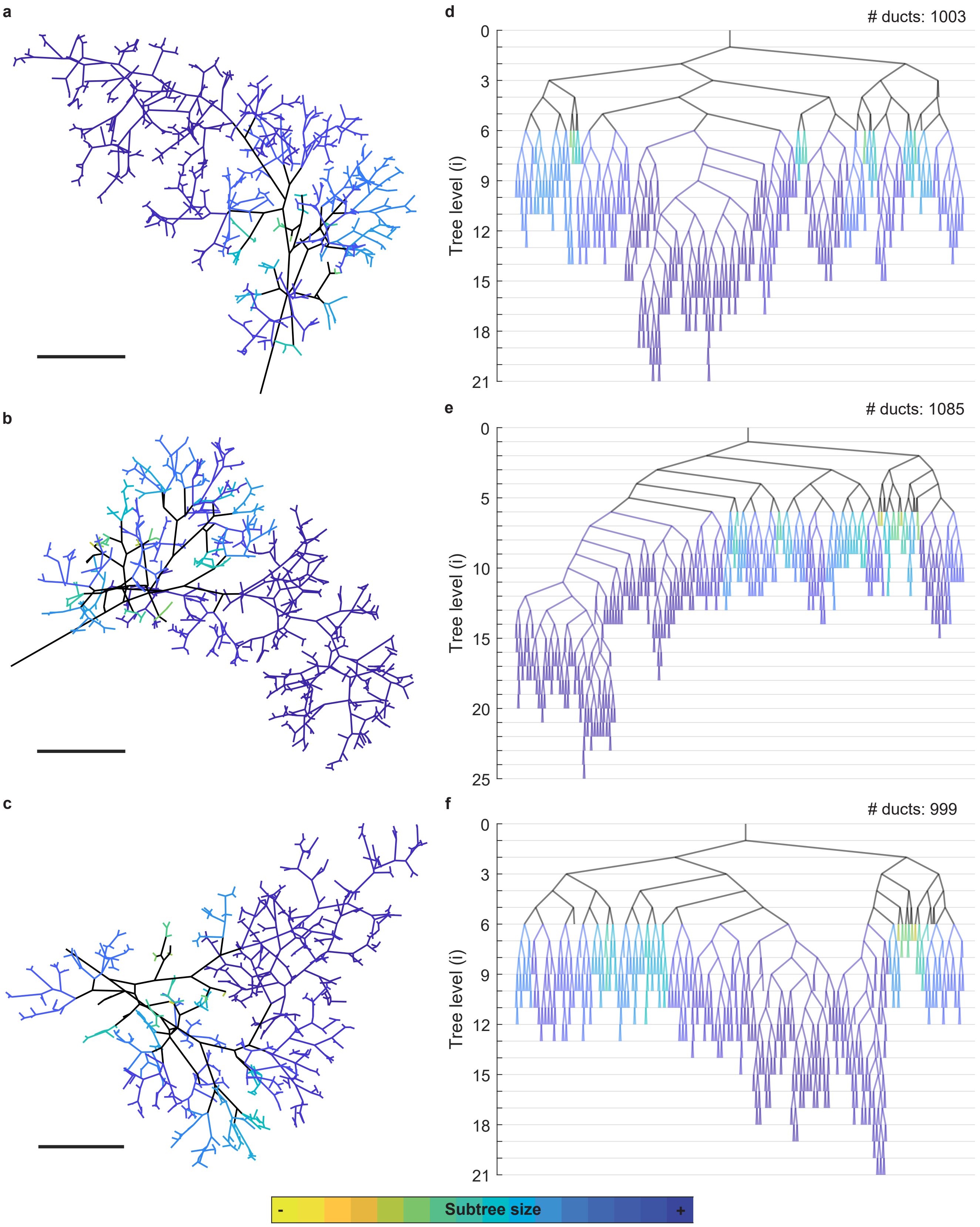

FIG. 4: **Reconstruction of first lobe of the mouse SMG at E16.5. a-c**, XY-projection of the ductal organization of the first lobe of the mouse SMG obtained from segmentation of the 3d confocal images. Individual subtrees from branch level 6 are indicated by different colors. All scale bars correspond to 200μm. **d-f**, Branching trees corresponding to the projections shown in (a-c), indicating the number of ducts in the tree, where subtrees are colored using the same color-code as in (a-c), as indicated by the color bar (bottom).

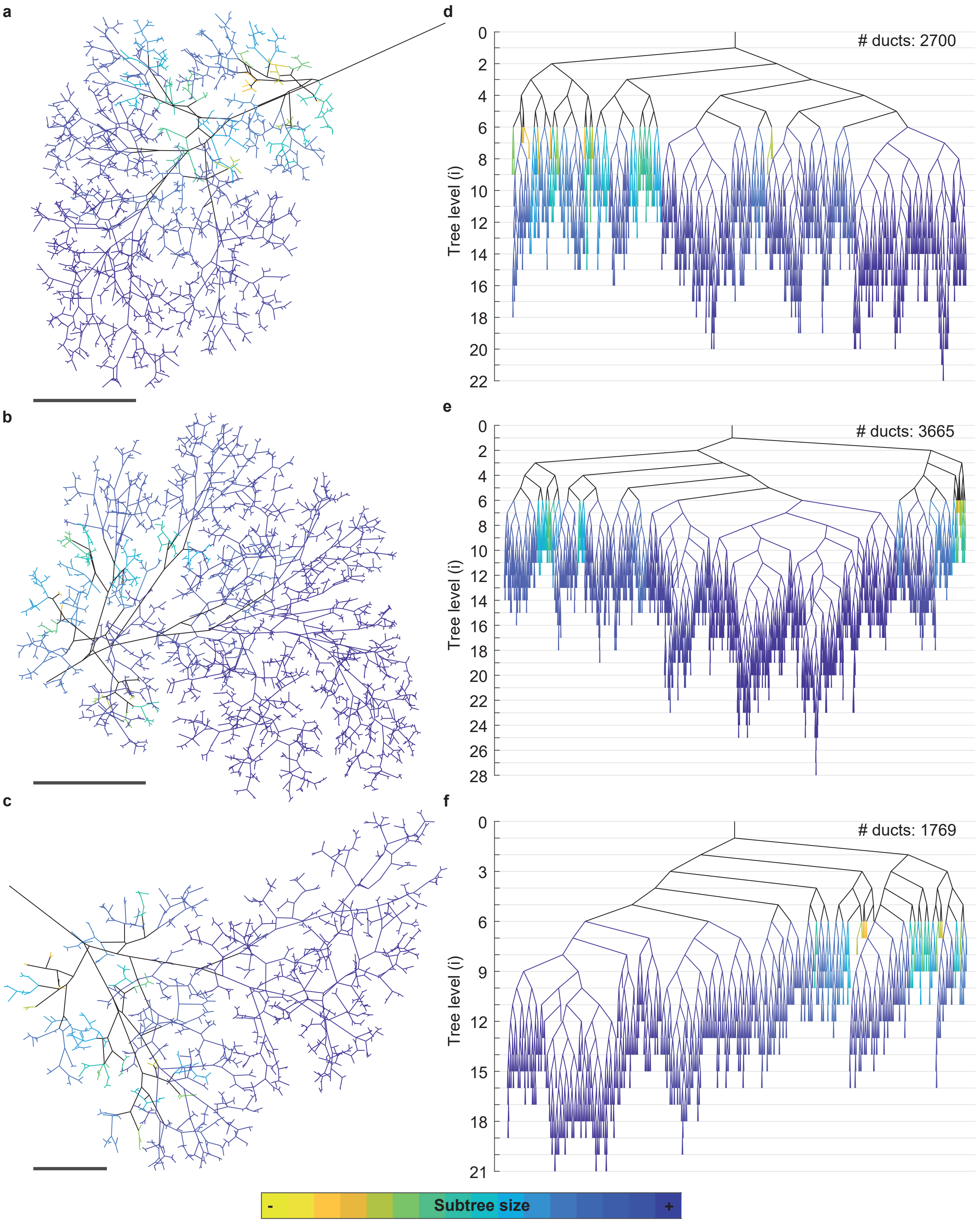

FIG. 5: **Reconstruction of first lobe of the mouse SMG at E18.5. a-c**, XY-projection of the ductal organization of the first lobe of the mouse SMG obtained from segmentation of the 3d confocal images. Individual subtrees from branch level 6 are indicated by different colors. All scale bars correspond to 200μm. **d-f**, Branching trees corresponding to the projections shown in (a-c), indicating the number of ducts in the tree, where subtrees are colored using the same color-code as in (a-c), as indicated by the color bar (bottom).

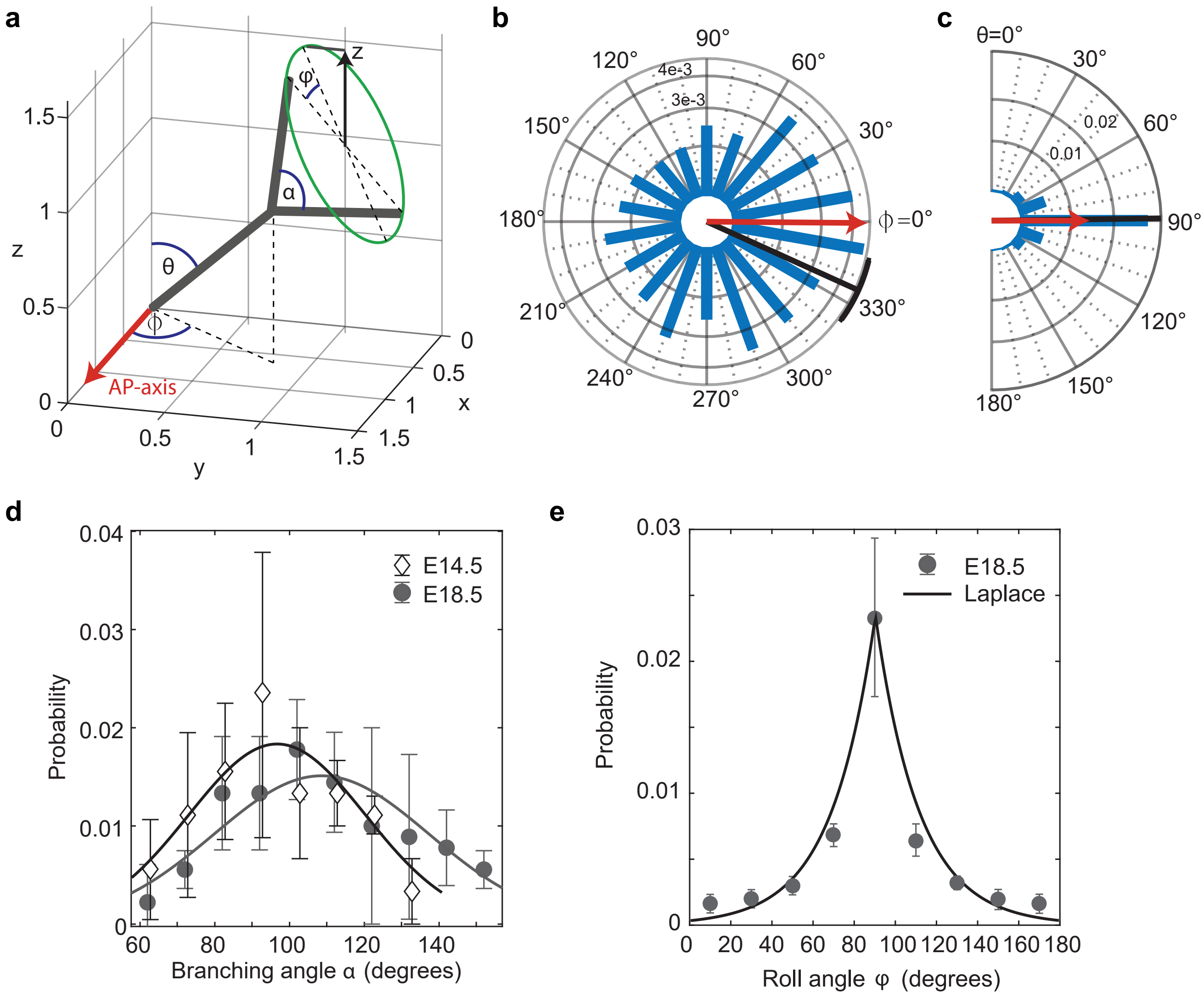

FIG. 6: **Statistics of ductal branching.** **a**, Schematic showing the angular parameters of a duct and its offspring following a bifurcation event, where the main duct (A-P axis) is oriented along $\phi = 0$ and $\theta = \pi/2$, as indicated by the red arrow in (a)-(c). **b**,**c**, Polar plot showing the distribution of branching angles of (b) ϕ and (c) θ, as defined in panel (a). Mean and SD are shown in black. **d**, Distribution of branching angles α at E14.5 and E18.5. Points show mean and SD. Parameterization used for stochastic simulations is shown by the continuous lines for E14.5 (diamonds) and E18.5 (circles). **e**, Distribution of roll angles, φ, showing an empirical fit (mean and SD) to a Laplace distribution, Eq. (S3). All measurements were made based on $n = 3$ biological replicates.

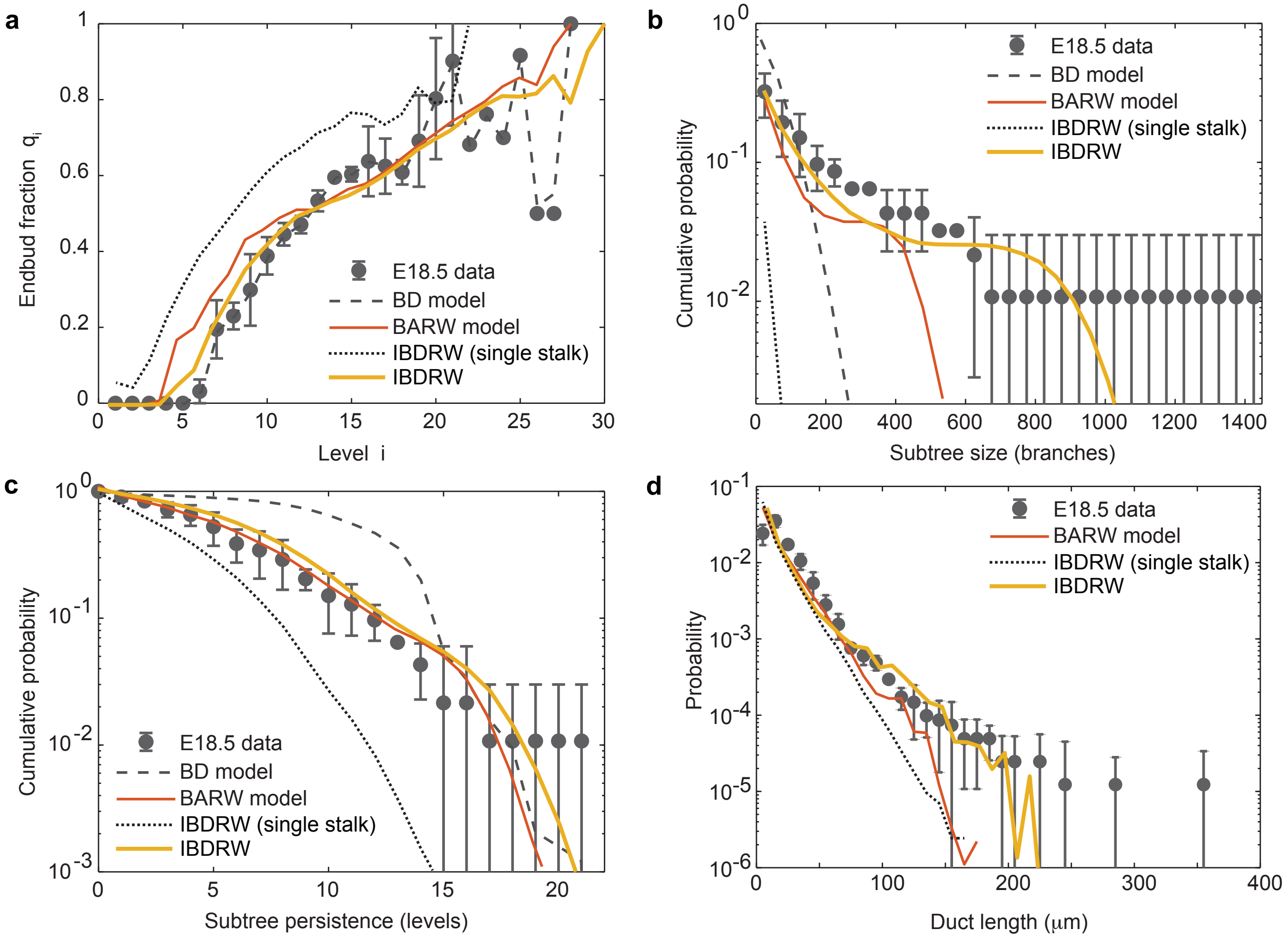

FIG. 7: **Fits to the branching statistics of the SMG based on alternative branching models.** **a**, Branch termination probability, q_i (BARW: $R^2 = 0.79$, IBDRW: $R^2 = 0.88$). **b-c**, Cumulative distribution of (b) subtree size (BARW: $R^2 = 0.43$, IBDRW: $R^2 = 0.96$) and (c) subtree persistence (BARW: $R^2 = 0.99$, IBDRW: $R^2 = 0.99$). **d**, Probability distribution of duct lengths (BARW: $R^2 = 0.63$, IBDRW: $R^2 = 0.88$). Experimental data from n=3 biological repeats (presented as mean values ± SD), and fit to the IBDRW model (reproduced from Fig. 3c-3f), are shown alongside corresponding fits to the standard BARW model (red line), and a zero-dimensional birth-death (BD) model (dashed black line). Here, for the IBDRW model, the initial condition involved a single stalk (IBDRW (single stalk) or the E14.5 rudimentary tree (IBDRW). For the BARW model, the results are obtained from a stochastic simulation with parameters $r_{\rm branch} = 0.45$ and $\rho_{\rm a} = 45$ μm and an initial condition translating to the rudimentary tree obtained empirically at the E14.5 time point. (For details on the stochastic simulations and fits, see Supplementary Note.) For the BD model, branching and termination events are considered as purely probabilistic, given by the empirical measurements of the endbud fraction $q_i(t)$ (see Supplementary Note). In both comparisons, statistics were obtained from 1000 realizations of the stochastic simulation. Panel (d) does not show data for the BD model, as this is a zero-dimensional model, and hence it does not account for the spatial growth of ducts. For simulations of the IBDRW (single stalk) we used the parameters in Table S1, with a single stalk as initial conditions and a simulations time of 156 hours, to account for the 6.5 days from E14.5 to E18. We note that the tail of the cumulative distributions of subtree sizes (b), which is not captured by any of the models, corresponds to a single large subtree (see large dark blue subtree in Supplementary Fig. 5e) belonging to the sample with the most branching generations (28, compared to 22 and 21 in samples 1 and 3). This sample-to-sample variability could originate from small differences in the organ size and/or developmental time between different mice. Additionally, the long duct in the tail of the distribution of duct lengths (d), correspond to primary ducts of the samples, which are generally long and thick.

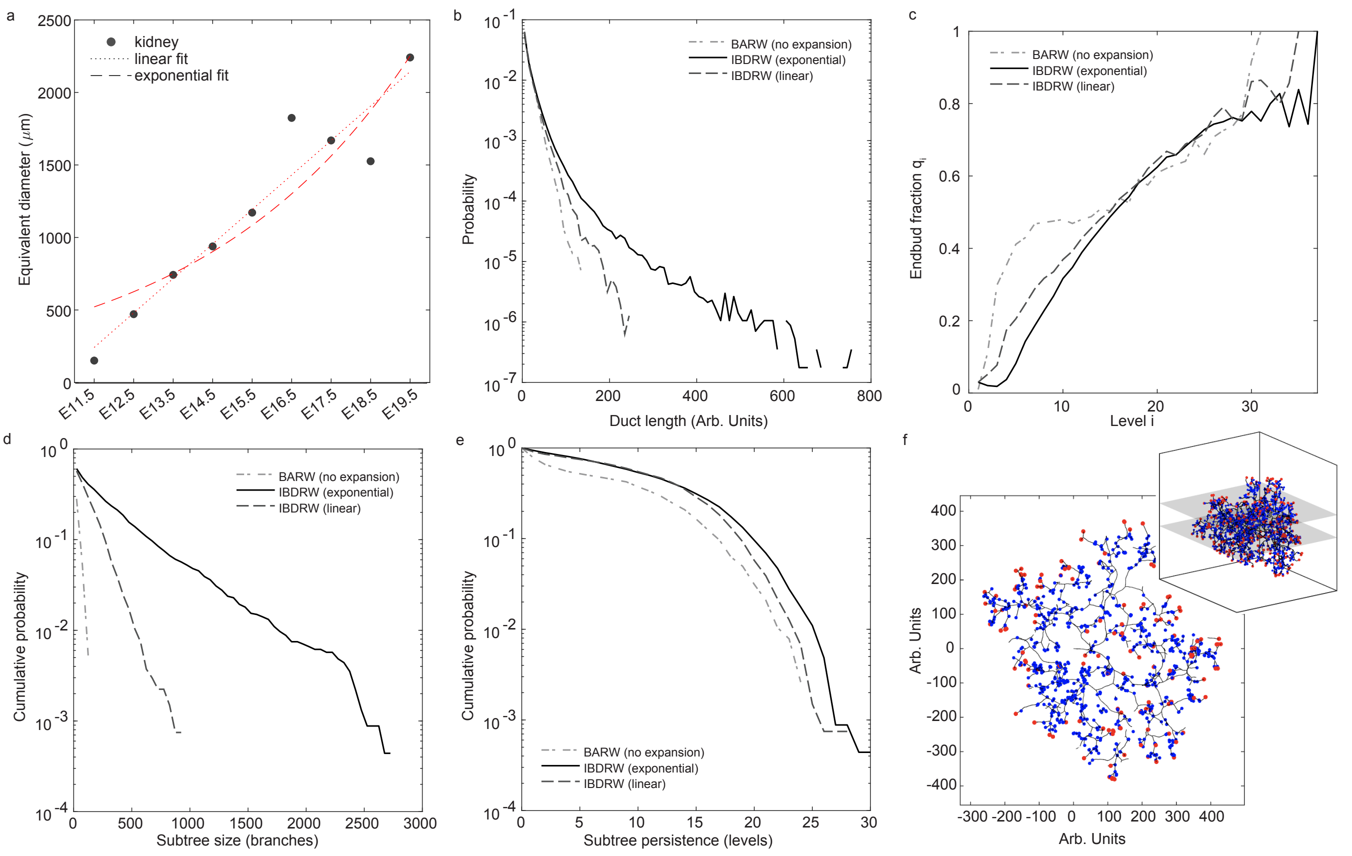

FIG. 8: **Long term effect of linear expansion on the branching dynamics. a**, Measurements of the effective diameter of the kidney throughout development, obtained from the 2d-projections in Sampogna et al. [14], exhibit a linear-like growth characteristic, with a fitted linear growth speed of 10μm/h ($R^2 = 0.91$), with an exponential growth curve producing a much poorer fit to the data ($R^2 = 0.84$). **b-e**, Statistics of (b) distribution of duct lengths, (c) endbud fraction, (d) subtree size, and (e) subtree persistence obtained from the standard BARW model and the IBDRW model with either linear or exponential inflation, with parameters as shown in Table S1, considering a single stalk as initial condition, a simulation time equivalent to 300 hours, and uniformly distributed branching angles φ (Supplementary Fig. 6a), resulting in an isotropic network. The expansion rate for the exponential inflation was set to $R_{\text{exp}} = 0.05$ h^{-1} while, for linear growth, the expansion was proportional to time, with the same constant of proportionality. **f**, Realisation of the IBDRW with linear inflation (inset), and a projection of a 2d section along the center of the network, with active tips highlighted in red and inactive tips shown in blue. Note that active tips localized predominantly at the periphery.

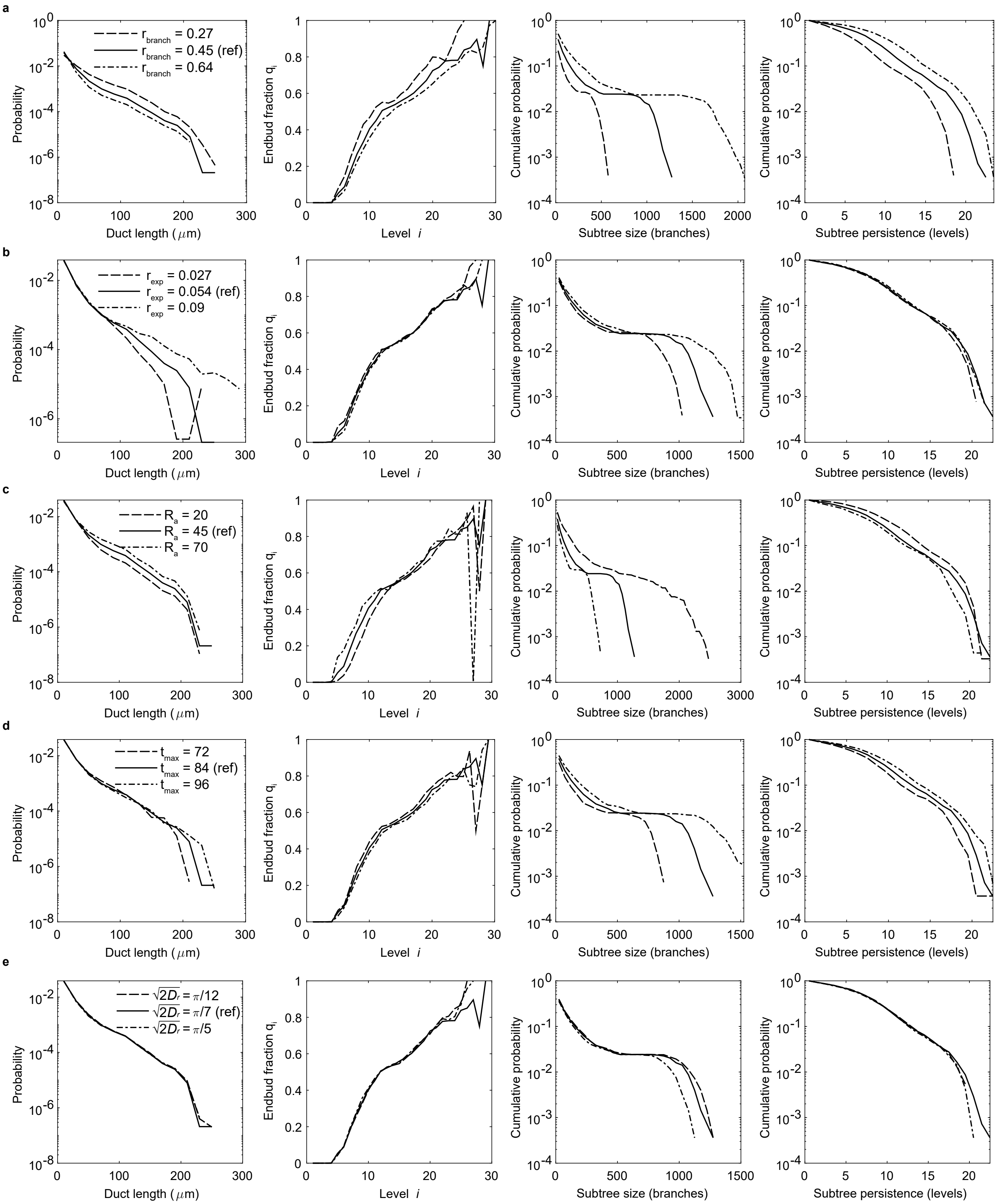

FIG. 9: **Sensitivity of the IBDRW model predictions to model parameters. a-e**, From left to right, sensitivity of duct length distribution, endbud fraction, subtree sizes and subtree persistence predicted by the IBDRW to changes in (a) branching rate, (b) expansion rate, (c) inhibition radius, (d) simulation time, and (e) rotational diffusion, as indicated. The reference (ref) curve (solid line) shows the results of the model using the parameters in Table S1. For each case, only the indicated parameter was varied.

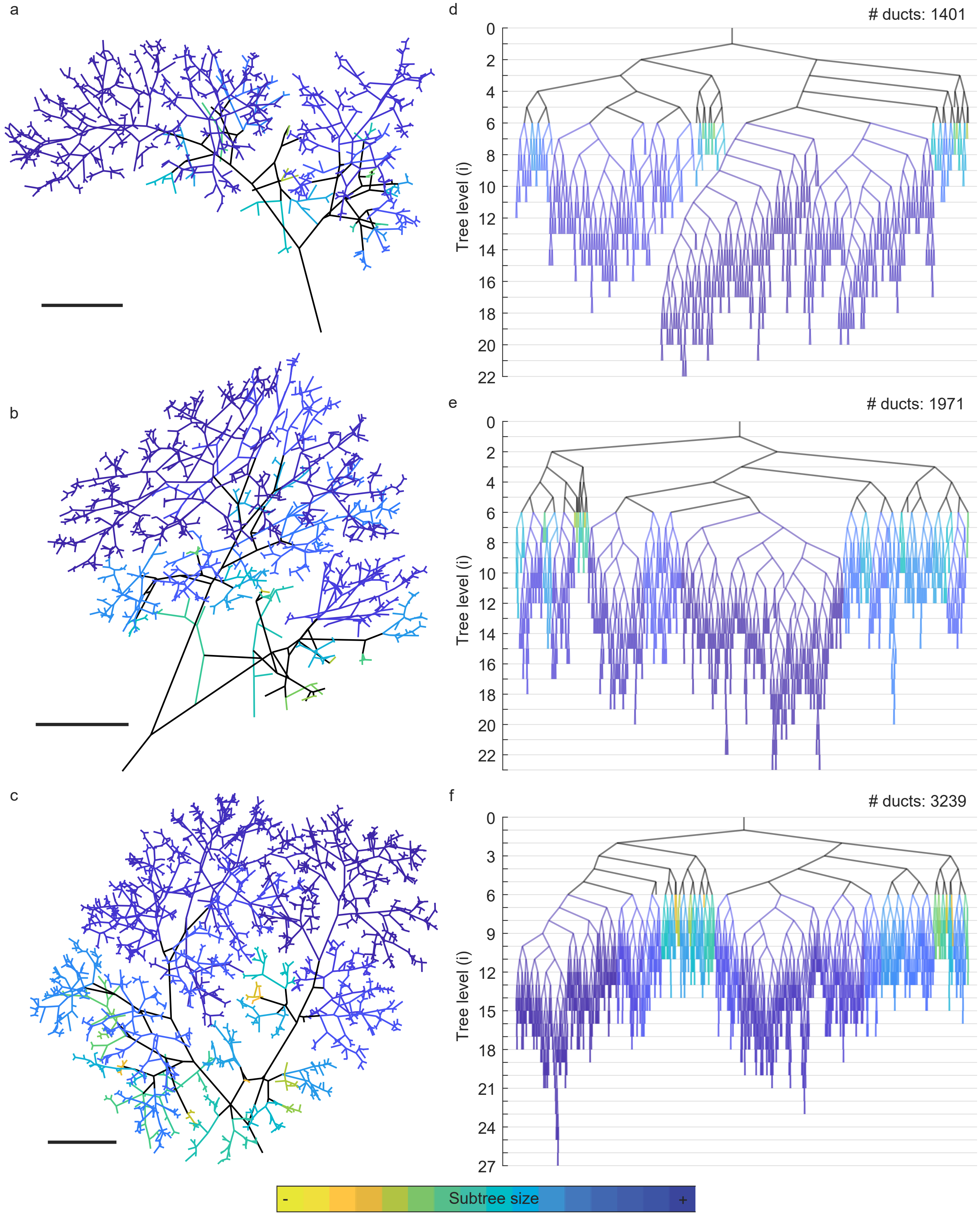

FIG. 10: **Reconstruction of explants of the mouse SMG harvested at E14.5 and cultured for 3 days.** **a-c**, XY-projection of the ductal organization of explants the mouse SMG obtained from segmentation of 3d confocal images. Individual subtrees from branch level 6 are indicated by different colors. All scale bars correspond to 200μm. **d-f**, Branching trees corresponding to the projections shown in (a-c), indicating the number of ducts in the tree, where subtrees are colored using the same color-code as in (a-c), as indicated by the color bar (bottom).

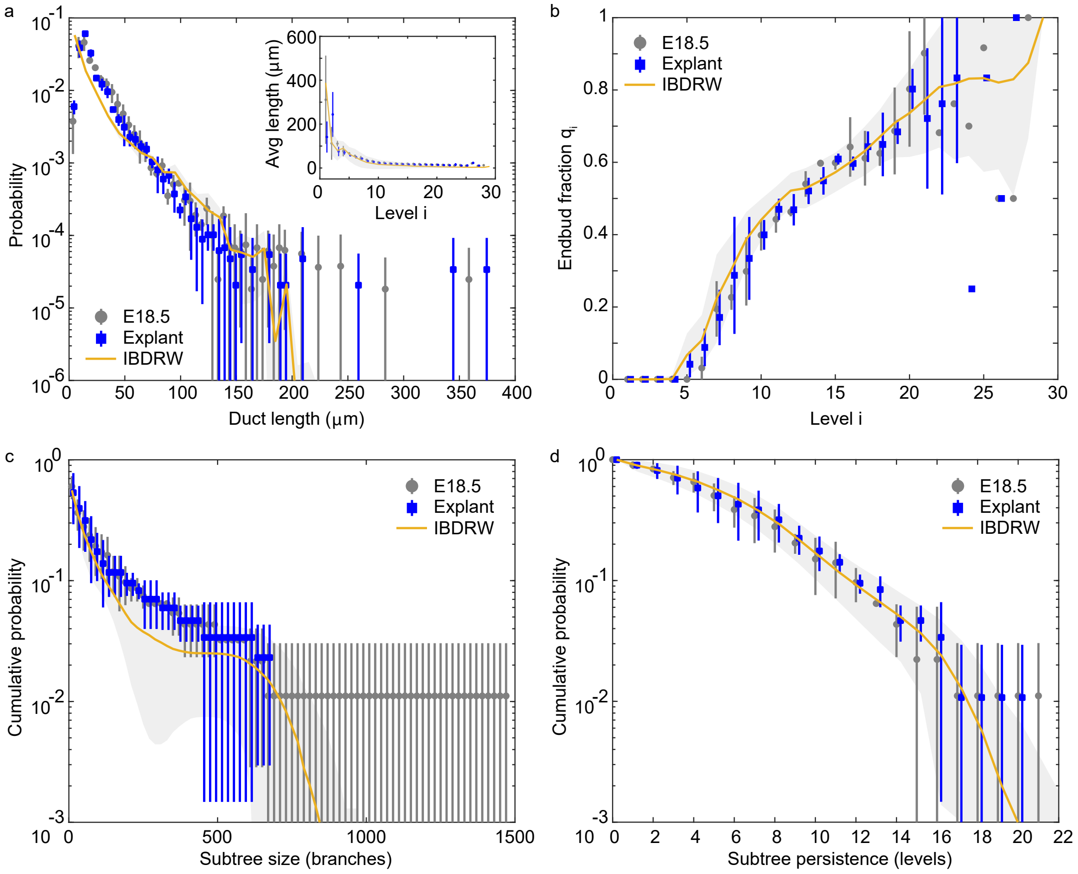

FIG. 11: **Branching statistics of explants harvested at E14.5 and cultured for 3 days. a-d**, Statistical distribution of (a) duct lengths and (inset) average duct length as a function of branch level, (b) endbud fraction, (c) subtree size, and (d) subtree persistence for explant culture (blue) compared to the E18.5 *in vivo* data (gray markers) and the IBDRW model extrapolated to the E14.5 + 3 day time point (average shown in yellow and SD shaded in light gray obtained from $n = 10^3$ realisations of the stochastic simulation).

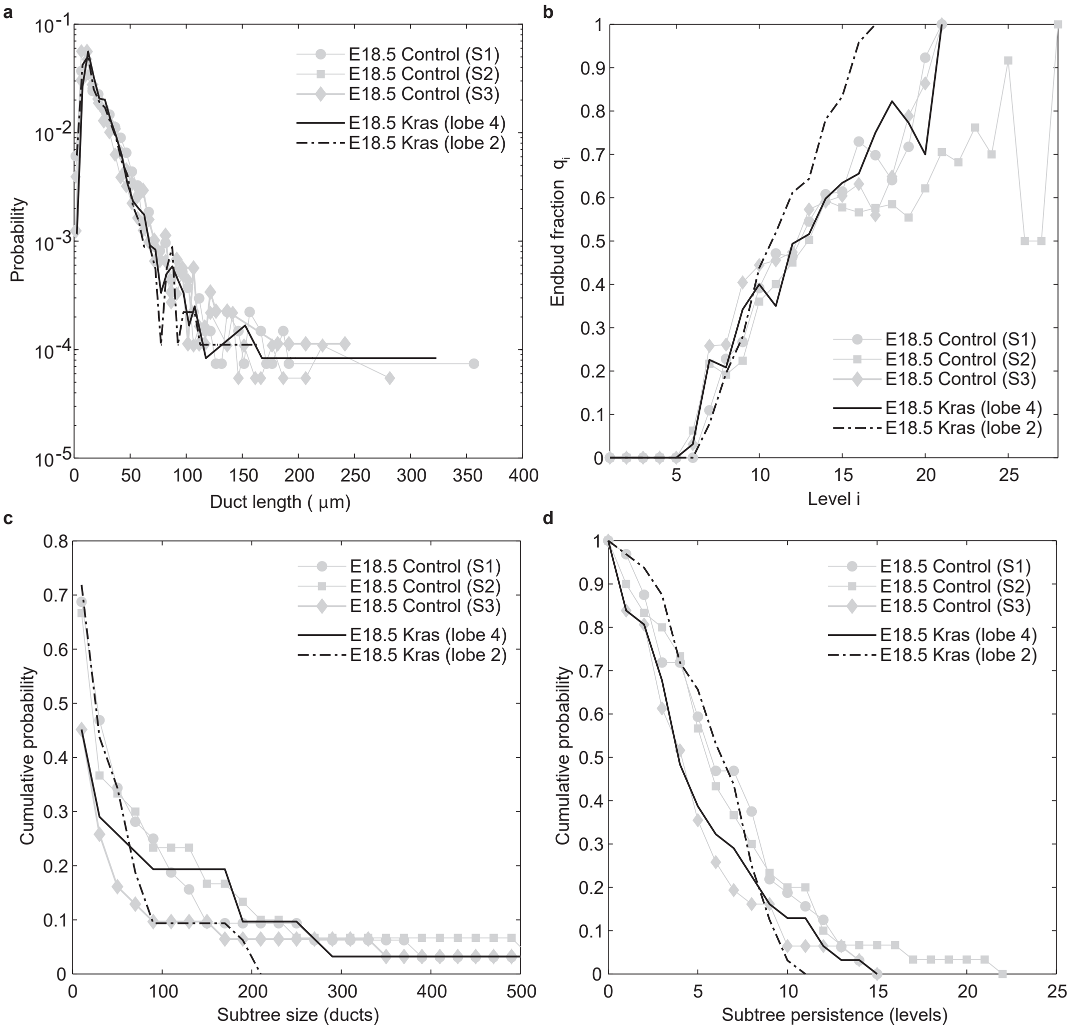

FIG. 12: **Branching statistics of lobes 2 and 4 of the mouse SMG. a-d**, Statistical distribution of (a) duct lengths, (b) endbud fraction, (c) subtree size, and (d) subtree persistence for the three control samples (all from lobe 1) and $n = 2$ samples from lobes 2 and 4. Note that, here, data was collected from red2Kras confetti mice induced at (low) clonal density with a small minority of RFP+ labelled cells mutant for Kras (see Supplementary Note).
