# Supplementary material for: Inflationary theory of branching morphogenesis in the mouse salivary gland
Source: Nat Commun. 2023 Jun 9;14:3422. doi: 10.1038/s41467-023-39124-x (PMC10256724; doi:10.1038/s41467-023-39124-x)
Supplement: Supplementary file 2 — Description of Additional Supplementary Files [file 41467_2023_39124_MOESM2_ESM.pdf]

Description of Additional Supplementary Files

Supplementary movie 1: Live-imaging of an mTmG E14.5 salivary gland explant cultured for 2 days. Data was acquired in time-lapse with images taken every hour (Methods). Endbuds that become submerged within the growing tissue exhibit a lower branching capacity than those positioned at the periphery (see Supplementary Fig. 1).

Supplementary movie 2: Numerical simulation of the (non-inflating) BARW model. Left, simulation of the BARW model for parameters rbranch = 0.45 and ρa = 45μm, with a simulation time of 84 hours, taking as an initial condition the branching network at E14.5. Active tips are marked in red and delayed tips in blue. Right, (top) branching tree and (bottom) endbud fraction obtained from the simulations (left). The nonexpanding domain causes submerged tips to terminate irreversibly, as shown by the large majority of blue (delayed) tips in the central region of the gland.

Supplementary movie 3: Numerical simulation of the IBDRW model. Left, numerical simulation of the IBDRW model (shown in Fig. 3b) for parameters rbranch = 0.45, rexp = 0.054 and ρa = 45μm, with a simulation time of 84 hours, taking as an initial condition the branching network at E14.5. Active tips are marked in red and delayed tips in blue. Right, (top) branching tree and (bottom) endbud fraction obtained from the simulations.
