# Supplementary material for: Inflationary theory of branching morphogenesis in the mouse salivary gland
Source: Nat Commun. 2023 Jun 9;14:3422. doi: 10.1038/s41467-023-39124-x (PMC10256724; doi:10.1038/s41467-023-39124-x)
Supplement: Supplementary file 6 — Reporting Summary [file 41467_2023_39124_MOESM6_ESM.pdf]

nature portfolio

Corresponding author(s): Benjamin D. Simons

Last updated by author(s): 2023/03/01

Reporting Summary

Nature Portfolio wishes to improve the reproducibility of the work that we publish. This form provides structure for consistency and transparency in reporting. For further information on Nature Portfolio policies, see our Editorial Policies and the Editorial Policy Checklist.

Statistics

For all statistical analyses, confirm that the following items are present in the figure legend, table legend, main text, or Methods section.

n/a	Confirmed	
☐	☒	The exact sample size (*n*) for each experimental group/condition, given as a discrete number and unit of measurement
☐	☒	A statement on whether measurements were taken from distinct samples or whether the same sample was measured repeatedly
☐	☒	The statistical test(s) used AND whether they are one- or two-sided<br>*Only common tests should be described solely by name; describe more complex techniques in the Methods section.*
☐	☒	A description of all covariates tested
☐	☒	A description of any assumptions or corrections, such as tests of normality and adjustment for multiple comparisons
☐	☒	A full description of the statistical parameters including central tendency (e.g. means) or other basic estimates (e.g. regression coefficient) AND variation (e.g. standard deviation) or associated estimates of uncertainty (e.g. confidence intervals)
☐	☒	For null hypothesis testing, the test statistic (e.g. *F*, *t*, *r*) with confidence intervals, effect sizes, degrees of freedom and *P* value noted<br>*Give P values as exact values whenever suitable.*
☒	☐	For Bayesian analysis, information on the choice of priors and Markov chain Monte Carlo settings
☒	☐	For hierarchical and complex designs, identification of the appropriate level for tests and full reporting of outcomes
☒	☐	Estimates of effect sizes (e.g. Cohen's *d*, Pearson's *r*), indicating how they were calculated

Our web collection on statistics for biologists contains articles on many of the points above.

Software and code

Policy information about availability of computer code

Data collection	MATLAB (R2022b). Natick, Massachusetts: The MathWorks Inc.<br>LAS software v2.8.0, Leica
Data analysis	Fiji (ImajeJ2 v2.9.0/1.53t)<br>MATLAB (R2022b). Natick, Massachusetts: The MathWorks Inc.<br>Custom scripts are available on the following Github repository: https://github.com/ibordeu/scripts_IBDRW_salivary_gland_git.

For manuscripts utilizing custom algorithms or software that are central to the research but not yet described in published literature, software must be made available to editors and reviewers. We strongly encourage code deposition in a community repository (e.g. GitHub). See the Nature Portfolio guidelines for submitting code & software for further information.

Data

Policy information about availability of data

All manuscripts must include a data availability statement. This statement should provide the following information, where applicable:

- Accession codes, unique identifiers, or web links for publicly available datasets
- A description of any restrictions on data availability
- For clinical datasets or third party data, please ensure that the statement adheres to our policy

Source data are provided as a Source Data file

Human research participants

Policy information about studies involving human research participants and Sex and Gender in Research.

Reporting on sex and gender	n/a
Population characteristics	n/a
Recruitment	n/a
Ethics oversight	n/a

Note that full information on the approval of the study protocol must also be provided in the manuscript.

Field-specific reporting

Please select the one below that is the best fit for your research. If you are not sure, read the appropriate sections before making your selection.

☒ Life sciences ☐ Behavioural & social sciences ☐ Ecological, evolutionary & environmental sciences

For a reference copy of the document with all sections, see nature.com/documents/nr-reporting-summary-flat.pdf

Life sciences study design

All studies must disclose on these points even when the disclosure is negative.

Sample size	No sample size calculation was performed.<br>We considered n=3 biological repeats per experimental time-point, as they provided enough statistical information to challenge and discern between distinct theoretical models.
Data exclusions	No data were excluded.
Replication	We considered data from multiple mice and different conditions (in supplementary material) in order to challenge and validate the model predictions.
Randomization	Randomization is not relevant for this study, as we were not comparing sample groups.
Blinding	Blinding was not applied, as we were not comparing sample groups.

Reporting for specific materials, systems and methods

We require information from authors about some types of materials, experimental systems and methods used in many studies. Here, indicate whether each material, system or method listed is relevant to your study. If you are not sure if a list item applies to your research, read the appropriate section before selecting a response.

Materials & experimental systems

n/a	Involved in the study	
☐	☒	Antibodies
☒	☐	Eukaryotic cell lines
☒	☐	Palaeontology and archaeology
☐	☒	Animals and other organisms
☒	☐	Clinical data
☒	☐	Dual use research of concern

Methods

n/a	Involved in the study	
☒	☐	ChIP-seq
☒	☐	Flow cytometry
☒	☐	MRI-based neuroimaging

Antibodies

Antibodies used	Primary: Rabbit anti-laminin (L9393, Sigma). Rabbit anti-Muc1 (ab15481, Abcam), Rabbit anti-Mist1 (ab187978, Abcam) and anti-Beta-catenin (L54E2) Alexa Fluor 488 Conjugate (#2849, Cell Signalling).<br>Secondary: Alexa Fluor 647 Donkey Anti-Rabbit (A31573, Thermo Fisher Scientific).
Validation	Anti-laminin (L9393, Sigma): Independent Antibody Verification – Demonstrating antibody specificity through the use of multiple

Validation

antibodies against target in IHC or ICC.
https://www.sigmaaldrich.com/GB/en/product/sigma/l9393

Anti-Muc1 (ab15481, Abcam): The manufacturer does not provide validation information. However, staining of MCF-7 cell line with the antibody ab15481 and another Anti-Muc1 validated antibody (ab218998) produced similar results.
https://www.abcam.com/products/primary-antibodies/muc1-antibody-ab15481.html?productWallTab=ShowAll#lb
https://www.abcam.com/products/primary-antibodies/muc1-antibody-ep1024y-low-endotoxin-azide-free-ab218998.html#lb

Anti-Mist1 (ab187978, Abcam): Over-expression Validation – Specific staining of the antibody in HEK-293T cells over-expressing Mist1 but not in HEK-293T cells transfected with vector only.
https://www.abcam.com/products/primary-antibodies/mist-1-antibody-ab187978.html?productWallTab=ShowAll

anti - Beta-catenin (L54E2) Alexa Fluor 488 Conjugate (#2849, Cell Signalling): The manufacturer does not provide validation information. However, staining of HeLa cells with the antibody b-catenin (L54E2) #2849 and another validated anit-b-catenin antibody (ab16051) produced similar results.
https://www.cellsignal.com/products/antibody-conjugates/b-catenin-l54e2-mouse-mab-alexa-fluor-488-conjugate/2849
https://www.abcam.com/products/primary-antibodies/beta-catenin-antibody-ab16051.html#lb

Animals and other research organisms

Policy information about studies involving animals; ARRIVE guidelines recommended for reporting animal research, and Sex and Gender in Research

Laboratory animals

Mouse, C57BL/6 background, The embryonic stages used were of E14.5, E16.5 and E18.5. Adult females of 6-20 weeks of age and males of 6-32 weeks of age were used for breeding.
Mice were kept in a pathogen-free facility under a 12-hour light and dark cycle.
Food and water were provided ad libitum.
Room temperature was maintained at 22°C ± 1°C with 30–70% humidity.

Wild animals

The study did not involve wild animals.

Reporting on sex

Since there is no sexual dimorphism at the embryonic stage, we did not distinguish between males and females.

Field-collected samples

The study did not involve samples collected from the field.

Ethics oversight

All experiments were performed according to the Home Office regulations and approved by the University of Cambridge Animal Welfare and Ethical Review Body.

Note that full information on the approval of the study protocol must also be provided in the manuscript.
